# The American Society for Microbiology’s evidence-based laboratory medicine practice guidelines for the diagnosis of bloodstream infections using rapid tests: a systematic review and meta-analysis

**DOI:** 10.1128/cmr.00137-24

**Published:** 2025-06-16

**Authors:** Donna M. Wolk, J. Scott Parrott, N. Esther Babady, A. Brian Mochon, Ryan Tom, Christen Diel, Jennifer Dien Bard, Amanda Harrington, D. Jane Hata, Amity L. Roberts, Lindsay Boyce, J. Kristie Johnson

**Affiliations:** 1 Geisinger and Geisinger Commonwealth School of Medicine 150362 , Danville and Scranton, Pennsylvania, USA; 2 Department of Interdisciplinary Studies, Department of Biostatistics and Epidemiology, School of Health Professions, School of Public Health, Rutgers, The State University of New Jersey 242612, New Brunswick, New Jersey, USA; 3 Department of Pathology and Laboratory Medicine, Memorial Sloan Kettering Cancer Center 5803, New York, New York, USA; 4 Departments of Pathology and Child Health, College of Medicine-Phoenix, University of Arizona, Phoenix, Arizona, USA; 5 Division of Pathology and Laboratory Medicine, Phoenix Children’s Hospital, Phoenix, Arizona, USA; 6 Garnet Health Medical Center Catskills and The State University of New Jersey, New Brunswick, New Jersey, USA; 7 Wellstar MCG Health and The State University of New Jersey, New Brunswick, New Jersey, USA; 8 Department of Pathology and Laboratory Medicine, Children's Hospital Los Angeles, Keck School of Medicine, University of Southern California 12223, Los Angeles, California, USA; 9 Department of Pathology and Laboratory Medicine, Loyola University Medical Center 25815, Maywood, Illinois, USA; 10 Department of Laboratory Medicine and Pathology, Mayo Clinic Alix School of Medicine, Mayo Clinic in Florida 156400, Jacksonville, Florida, USA; 11 Department of Laboratory Medicine, Hartford HealthCare, Newington, Connecticut, USA; 12 Department of Research Informatics, Memorial Sloan Kettering Cancer Center 5803, New York, New York, USA; 13 Department of Pathology, University of Maryland School of Medicine 12264, Baltimore, Maryland, USA; Rush University Medical Center, Chicago, Illinois, USA; Rutgers Robert Wood Johnson Medical School, New Brunswick, New Jersey, USA

**Keywords:** bloodstream infection, blood culture, diagnostics, rapid tests, mass spectrometry, nucleic acid amplification test, medical outcomes, stewardship, guidelines

## Abstract

Bloodstream infections (BSIs) are a significant cause of mortality and morbidity. Rapid identification of pathogens and detection of a few resistance markers from positive blood cultures are now possible through the increased availability of commercial rapid diagnostic tests, including nucleic acid amplification tests and matrix-assisted laser desorption ionization time-of-flight mass spectrometry. This document describes the clinical utility of rapid diagnostics performed on positive blood cultures and provides evidence-based laboratory medicine guidelines for using rapid tests to diagnose BSIs in hospitalized adult and pediatric patients. This guideline was developed for use by medical (a.k.a. clinical) microbiologists, medical laboratory professionals, infectious disease clinicians, pharmacists, hospital administrators, healthcare providers, and other stakeholders associated with BSIs. A panel of experts, including medical microbiologists and experts in systematic literature review, was assembled to formulate the Population–Intervention–Comparison–Outcome (PICO) question, review the literature, and provide recommendations for using rapid tests to diagnose BSI and improve patient outcomes. A comprehensive literature search of four electronic bibliographic databases (MEDLINE, Embase, CINAHL, and Cochrane) was conducted to identify studies with measurable outcomes. The panel followed a systematic process, which included a standardized methodology for rating the certainty of the evidence and strength of recommendations using the GRADE (Grading of Recommendations, Assessment, Development, and Evaluation) approach. The panel evaluated the literature to answer the question: Does using rapid diagnostic tests improve clinical outcomes in adult and pediatric patients hospitalized with a BSI? Peer-reviewed literature was available to address three outcomes, including time to targeted therapy, mortality, and length of hospital stay. In general, the quality of the evidence was low to moderate due to the paucity of controlled, randomized clinical trial studies. However, eight recommendations were made based on evidence derived from the systematic review of the published literature. To answer the PICO question, the expert committee recommended using rapid diagnostic tests combined with active communication to decrease the time to targeted therapy and length of stay (strong recommendation). While the strength of the evidence for the impact on mortality is low, the panel supports using rapid tests to impact these outcomes. A summary of the recommendations is listed in the Executive Summary, which includes a detailed description of the background, methods, evidence summary, and rationale that supports each recommendation in the full text.

## INTRODUCTION

Identifying pathogens and corresponding antimicrobial susceptibility results optimizes care for patients with bloodstream infections (BSIs). Several tests, including nucleic acids amplification tests (NAAT), hybridization tests, and matrix-assisted laser desorption ionization time-of-flight mass spectrometry, rapidly identify common bacteria and yeast directly from a positive blood culture. Some molecular methods include genetic markers associated with antibiotic resistance. Because strategies used for rapid diagnostic tests (RDTs) vary by laboratory, the American Society for Microbiology assembled an expert panel to review the existing literature describing the effectiveness of RDTs in decreasing the time-to-targeted therapy for hospitalized patients with BSIs (1). The review was published in 2016, but no recommendations were made due to insufficient evidence. With the increased availability and broader use of rapid diagnostics, a second expert panel was convened to assess the evidence that RDTs improve outcomes in adult and pediatric patients hospitalized with BSIs, provided that active communication occurs. Because of differences in global healthcare infrastructures, the published literature varies in the way active communication is described. Some examples include phone calls or texts to a provider or active communication (voice or digital) to pharmacists or microbiologists associated with antimicrobial stewardship programs (2) (https://www.cdc.gov/antibiotic-use/hcp/core-elements/hospital.html), diagnostic stewardship programs (3), or other active methods determined in collaboration with healthcare providers and stakeholders. Based on the results of a scoping review (4), the panel evaluated the effectiveness of rapid diagnostics and developed recommendations to improve three clinical outcomes: time-to-targeted therapy, length of hospital stay, and mortality.

BSIs that lead to sepsis impact patient mortality and morbidity across the globe (5–7). The epidemiology of sepsis ranges from neonatates to adults and the elderly for both community-onset and hospital-onset sepsis (8–10). Healthcare costs for sepsis patients are extraordinarily high (11–14). In 2008 (15), the Surviving Sepsis Campaign issued guidelines for processes that should occur within the first 1 to 6 hours of potentially septic patients presenting to emergency departments (16–18). In the USA, these guidelines inspired the Centers for Medicaid and Medicare (CMS) directives and The Joint Commission inspection checklists for US hospitals (19–21). Both initiatives aimed to define compliance with published sepsis bundles in Emergency Department settings.

In 2010, the World Health Organization (WHO) defined critical attributes of a blood culture collection related to the time of collection before antibiotics, blood culture contamination, blood volume, and other features that impact diagnostic accuracy (22). In contrast, little has been done to define and standardize actions or processes occurring after the sepsis bundles (i.e., after the first 1 to 6 hours after the suspicion of sepsis). From its onset in 2008 to the latest document in 2021, the only references to laboratory methods listed in the Surviving Sepsis Guidelines are the collection of blood cultures before antibiotics are prescribed and lactate testing (18). The gold standard for diagnosing pathogens in BSIs remains traditional blood cultures followed by pathogen identification and phenotypic antimicrobial susceptibility testing (AST). The advantage of blood culture is the unbiased testing approach, which means that prior knowledge of the infecting microorganism(s) is unnecessary. Most bacteria and yeasts can be detected within 24–48 hours, with some fastidious organisms like members of the HACEK [*Haemophilus parainfluenzae, Aggregatibacter* (*Haemophilus*) *aphrophilus, Aggregatibacter* (*Actinobacillus*) *actinomycetemcomitans, Cardiobacterium hominis, Eikenella corrodens,* and *Kingella kingae*] taking up to 5 days (23). The most recent data from the international SENTRY antimicrobial surveillance program (1997–2016) reported *Staphylococcus aureus* and *Escherichia coli* as the two most common pathogens recovered in BSI (~20% each), followed by *Klebsiella pneumoniae* (7.7%), *Pseudomonas aeruginosa* (5.3%), and *Enterococcus faecalis* (5.2%) (24). Several factors may affect the blood culture system’s pathogen recovery rates, including the administration of broad-spectrum antibiotics before collecting blood cultures (25) and, most importantly, the volume of blood collected (26, 27).

Based on biochemical reactions (e.g., oxidase, catalase), conventional pathogen identification methods require pure isolates from subcultures of positive blood cultures for pathogen identification and AST. After bacteria and yeast are detected in blood culture broth, an additional 48–72 hours is typically required to identify pathogens that are subcultured to agar and achieve AST profiles. Thus, culture-based identification and AST further delay the implementation of targeted antimicrobial therapy (28, 29).

Rapid identification of pathogens and a few resistance markers directly from positive blood cultures has been significantly enhanced by the increased availability of commercial rapid diagnostic tests, including NAATs, hybridization methods, and matrix-assisted laser desorption ionization time-of-flight mass spectrometry (MALDI-TOF MS). Most assays used for identification of pathogens from positive blood cultures are molecular methods in “sample-to-answer" formats with time to report (TTR) of less than 2 hours (30). While MALDI-TOF MS requires a positive blood culture or an isolate for testing, the method has been validated for testing directly on a positive blood culture for faster pathogen identification (31). Rapid identification of bloodstream pathogens represents laboratory practices aimed to support fast and actionable changes to antimicrobial therapy, either by escalating therapy to target multidrug-resistant organisms or de-escalating antimicrobial coverage to target the drug and microbe combination better and reduce toxicities caused by broad-spectrum antibiotics. Intuitively, rapid diagnostic tests can significantly reduce the time for pathogen identification and implementation of targeted therapy compared to conventional methods. However, these results become meaningless if the patient care team does not see them in a timely manner. Hence, active communication of the rapid results to the provider must be part of the overall process for these tests to have a material impact on patient outcomes, such as mortality, morbidity, hospital length of stay (LOS), antibiotic use, and healthcare expenses. The published literature varies widely when describing how active communication occurs. Some examples include phone calls to a provider, active communication (voice or digital) by pharmacists or microbiologists associated with antimicrobial stewardship programs (ASPs), diagnostic stewardship programs (DSPs), or other active methods determined in collaboration with healthcare providers and stakeholders (1, 32; https://www.cdc.gov/antibiotic-use/hcp/core-elements/hospital.html).

The American Society for Microbiology (ASM) partnered with the Centers for Disease Control and Prevention (CDC) and Battelle consultants to perform a systematic review and meta-analysis intended to document the impact of the rapid identification of bloodstream pathogens on the downstream clinical outcomes for patients with BSIs (1). Although a trend was detected indicating that there may be some benefit for patients when the rapid identification of genus and species occurred and was reported to providers with authority for tailoring antibiotics, there were insufficient published data for the team to create an official recommendation for evidence-based guidelines (1).

The purpose of this study was to refresh the body of knowledge and evaluate the current evidence for the effectiveness of rapid diagnostic practices to decrease the time to targeted therapy (TTT) and improve outcomes for hospitalized patients with BSIs. This manuscript represents the refresh of the literature and subsequent systematic review and meta-analysis driven by a scoping review (4) to identify manuscripts and answerable questions that could be developed into microbiology laboratory guidelines that could lead to expectations for accredited laboratories (see “Full summary of recommendations and supporting evidence,” below).

## KEY DEFINITIONS

### Rapid test

For this review, a rapid diagnostic test (RDT) was defined as any assay (e.g., NAAT, microarray technology, MALDI-TOF MS, hybridization, including peptide nucleic acid fluorescence *in situ* hybridization, etc.) where an aliquot from a positive blood culture broth is used to directly identify a bacteria or yeast, rather than relying on conventional culture and traditional biochemicals with or without automated culture-based instruments to identify the organism. The expectation for RDT is that the testing would occur when the blood cultures flagged positive, or if the laboratory was not staffed 24/7/365, the outcome would be reported only for the patients for whom the RDT was tested promptly after the positive flag.

### Intervention chain types

Details on intervention types are detailed elsewhere (4). Briefly, laboratory improvement processes regarding BSI detection and intervention are not a single intervention but a set of interventions chained together to create an improved process, i.e., intervention chains. In the previous scoping review, different types of intervention chains were identified: (i) comprehensive chains that included process improvements in pre-analytic procedures, organism identification, and communication methods, (ii) test-focused chains that were improvement interventions focused only on organism identification, and (iii) communication chains where the primary intervention entailed changes in the procedures by which rapid test results were reported to clinicians, pharmacists, or antimicrobial stewardship teams.

### TTT

Time to targeted therapy was defined as the duration (in hours) from when the rapid test identified the etiological agent from a positive blood culture to the initiation of appropriate or optimal antimicrobial therapy, according to the individual manuscript for which it was deployed.

### LOS

Length of stay was defined as the time (in days) between the patient’s admission and when they either were discharged from the facility or received a step-down in care (i.e., transfer from an intensive care setting to an inpatient ward). The scoping review identified three types of hospital stays: (i) LOS of patients with a positive blood culture, (ii) LOS in the intensive care unit (ICU) of patients with a positive blood culture, and (iii) infection-related LOS of patients with a positive blood culture, which measures the LOS during the bloodstream infection.

### Mortality

Mortality was defined as the number of deaths associated with patients having positive blood cultures. As part of the scoping review, four variants of mortality were identified: (i) non-specific mortality, (ii) 30-day mortality, (iii) in-hospital mortality, and (iv) infection-related mortality, which measured the mortality rate during the bloodstream infection. In-hospital mortality and non-specific mortality were used when the time frame was unclear.

## METHODS

A panel of experts that included medical microbiologists and experts in systematic literature review was assembled to formulate the Population–Intervention–Comparison–Outcome (PICO) question, review the literature, and provide recommendations for using rapid tests to diagnose BSI and improve patient outcomes. A comprehensive literature search of four electronic bibliographic databases (MEDLINE, Embase, CINAHL, and Cochrane) was conducted to identify studies with measurable outcomes. The panel followed a systematic process that included a standardized methodology for rating the certainty of the evidence (32) and strength of recommendations using the GRADE approach (Grading of Recommendations Assessment, Development and Evaluation) (33).

### Panel composition

The expert panel was selected to include medical microbiologists from across the USA with expertise in diagnosing BSIs using rapid diagnostic tests (D.M.W., N.E.B., A.B.M., J.D.B., A.H., D.J.H., A.L.R., J.K.J.). A core team of experts (D.M.W., N.E.B., A.B.M., J.K.J.) was selected by ASM and included a technical lead (J.K.J.) and a guideline coordinator (D.M.W.). The core team developed the PICO question and performed the initial literature review. The panel also included methodologists and experts in systematic literature review and guidelines development (J.S.P.). A librarian (L.B.) provided additional support for literature search, data extraction, and analysis (R.T., C.D., J.S.P.). Administrative support was provided by the ASM staff (N.J., P.M.).

### Search strategy

A comprehensive search was conducted in four databases in November 2016, and a refresh was performed in May 2019 to identify potentially relevant studies. The four databases MEDLINE (Ovid), Embase (Ovid), CINAHL (EBSCO), and Cochrane Central Register of Clinical Trials (Wiley) were searched for studies published from January 2011 through April 2019.

Animal studies and non-English publications were excluded. Controlled vocabulary and/or keyword searches were used in each of the four databases to develop search strategies. The search contained the following medical subject headings: bacteremia; bloodstream infection; time factors; healthcare costs; length of stay; morbidity; mortality; antimicrobial therapy; rapid molecular techniques, polymerase chain reaction (PCR); *in situ* hybridization, fluorescence; treatment outcome; drug therapy; patient care team; pharmacy service, hospital; hospital information systems; Gram stain; pharmacy service; and spectrometry, mass, matrix-assisted laser desorption-ionization. The word phenotypic was searched, as well as the following keywords: targeted therapy; rapid identification; rapid; Gram positive; Gram negative; reduce(d); cost(s); pneumoslide; PBP2; tube coagulase; matrix-assisted laser desorption/ionization time of flight; MALDI TOF; blood culture; EMR (electronic medical record); electronic reporting; call to provider; collaboration; pharmacy; laboratory; bacteria; yeast; ICU; and others. The library search strategies used Boolean operators and proximity searching to focus the search results. The results from each database were imported into EndNote, and duplicates were removed.

### Screening and study selection

**Fig 1:**
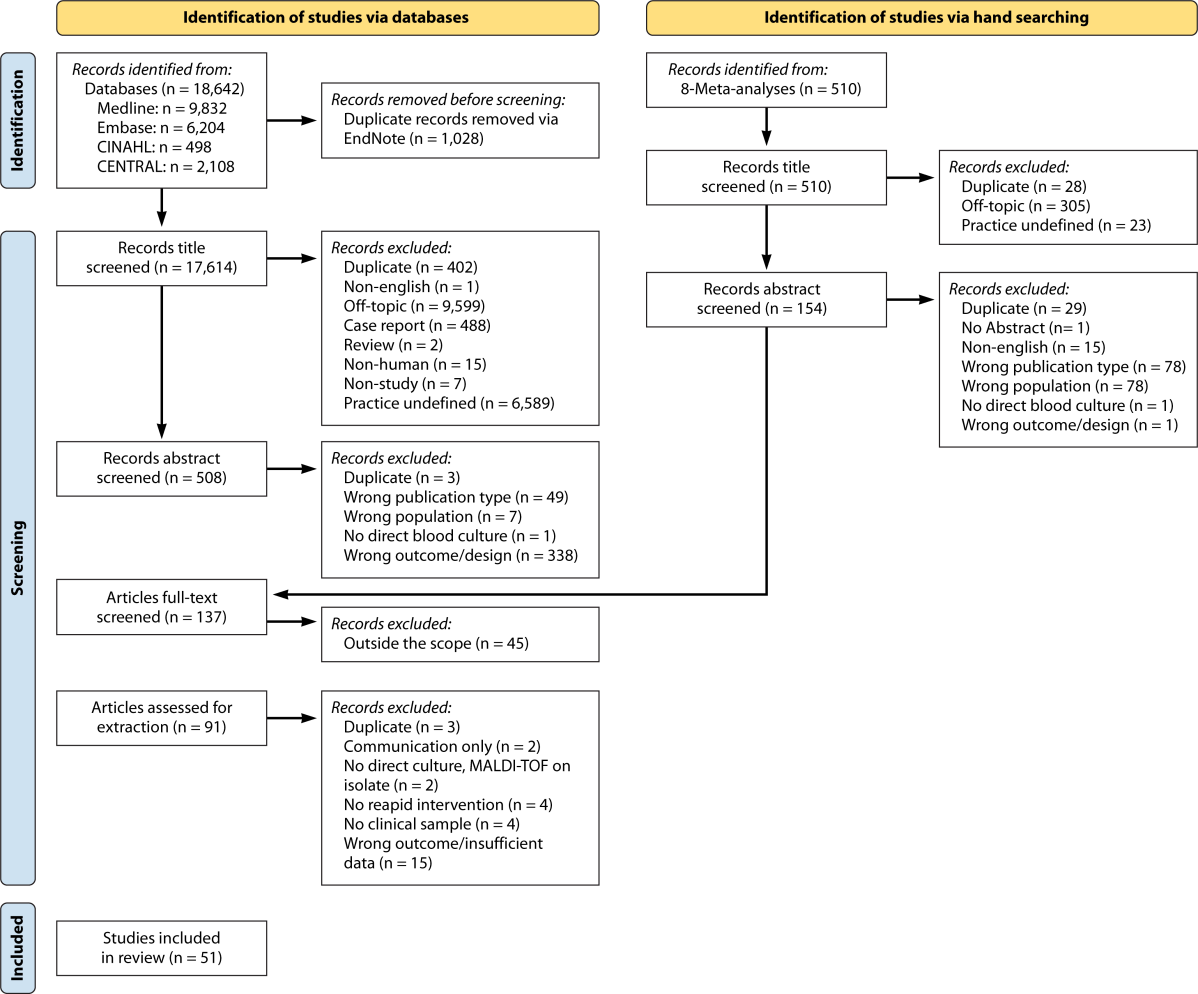
PRISMA flow diagram.

The technical lead (J.K.J.) and the guideline coordinator (D.M.W.) performed the initial screen and title review of all the identified studies. The studies were transferred into the Rayyan system (reference), and the core team (D.M.W., J.K.J., A.B.M., N.E.B.) reviewed the abstracts to identify relevant studies. Exclusion criteria included conference abstracts, reviews and meta-analyses, editorials, letters to the editor, testing performed on isolates only, testing with TTR of more than 8 hours, testing performed on non-human samples, and studies with no clinical outcomes or the wrong clinical outcomes (Fig. 1). The exact process was used for both the initial and literature refresh. All selected studies were subjected to data extraction as described below.

### Data extraction and collection

Data from extractions were captured within an extraction template in the US Agency for Healthcare Research and Quality’s Systematic Review Data Repository Plus (https://srdr.ahrq.gov/). The extraction template was created, and pilot testing was performed (34). Two expert panel members extracted all sources double-blind, and a third team member resolved conflicts. In addition to outcome information, data were also collected on study design details (e.g., study design, sampling strategy, funding source, and setting). Furthermore, arm details (using a framework based on a prior scoping review of the topic (4), including the details on test preparation, the organism identification and antimicrobial susceptibility test methods used, and the communication practices for test results) were included. Finally, sample characteristics (organisms identified, age, and sex of participants), results of the outcomes of interest (LOS, mortality, time to targeted therapy), and risk of bias were incorporated.

### Risk of bias assessment

Modified versions of the National Institute of Health Study Quality Assessment Tools were used for risk of bias (ROB) assessment (https://www.nhlbi.nih.gov/health-topics/study-quality-assessment-tools). Question wording and instructions included in the three ROB tools relevant to this project (before-after, cohort, and controlled intervention) were tailored to fit the context of the laboratory improvement process. To determine the overall ROB for each study, the team categorized questions into three domains (patient selection, intervention-related, outcome-related) and established critical flaw criteria relevant to laboratory improvement processes. An assessment algorithm was developed and then applied to each study. The core team reviewed individual study ROB ratings and reached a consensus on all studies. All studies were double-blinded and evaluated for ROB by expert panel members, with a third team member adjudicating any disagreement.

### Statistical methods

Three outcomes of interest were examined in this study: LOS, mortality, and TTT. For all models, random effects meta-analyses were used. The Paule-Mandel procedure was used to estimate Ƭ^2^ for binary and continuous outcomes due to its good performance across analysis scenarios (35). The mean differences were computed for pooled estimates of continuous outcomes (LOS and TTT), and for binary outcomes, risk ratios were calculated. R software packages {meta}, {metafor}, and {dmetar} were used for the analyses (https://dmetar.protectlab.org/). Refer to Fig. S1 for the algorithm that calculated the means and standard deviations.

#### Effect measures

For LOS and TTT, the mean and standard deviation of days (LOS) or hours (TTT) were extracted, and the change was reported as the mean difference (MD) between arms. The number of events per arm was extracted for mortality, and the change was reported as risk ratios. Several studies implemented the intervention effort in stages (e.g., the standard process for new organism identification test and then enhanced communication method), so outcomes were reported multiple times. For this analysis, only the last, most comprehensive stage of the implementation was used to compare to the baseline.

#### Synthesis eligibility, missing data, and data estimation

To be included in the analyses, target measures (e.g., means, SD, *n*) or non-normal measures of centrality and dispersion (e.g., median, 25th/75th percentiles, min/max) had to be reported in tables or narratives. Where data reported in studies were not present or sufficient to estimate the above effects (e.g., present only in figures), authors were contacted requesting additional information. Where the mean and SD were not available, the following procedures were used to estimate these measures from median and associated dispersion measures: the method proposed by Cai et al. (36) to adjust the estimates of those proposed by Luo et al. 2018 (37) for the mean and by Wan et al. (38) for the standard deviation. The Cai 2021 approach was used since the Luo 2016 and Wan 2014 approaches assume an underlying normal distribution (unrealistic for LOS and TTT). Cai 2021 allows for a Box-Cox transformation to normalize the data, so the Luo 2016 and Wan 2014 estimation approaches can be applied with a backward transformation as the final step. This procedure uses the R software package {estmeansd}. Examination of the estimates produced by this combination of procedures occasionally resulted in unrealistic estimates of the mean and SD (e.g., estimated means that fell above the value of the 75th percentile and standard deviations greater than twice the interquartile range). When estimates using the Cai 2021 approach fell outside these thresholds, the original Luo 2016 and Wan 2014 formulas were used (Fig. S1). A sensitivity analysis was carried out when the Luo 2016 or Wan 2014 methods were used to determine if the parameter estimation method affected the final pooled estimate of effect.

#### Diagnostics and reporting bias

Publication bias was assessed using funnel plots and Egger’s regression (39) for continuous outcomes and Peters’ test (40) for binary outcomes. Outliers and influential cases were screened using the R companion {dmetar} software package. Small study bias was assessed across all outcome types.

#### Heterogeneity and sensitivity analysis

Heterogeneity is reported using I2 (41). For mortality outcomes, separate analyses were performed based on the type of mortality reported: 30-day mortality, infection-related mortality, hospital mortality, and non-specific mortality where the time frame was unclear. Similarly, LOS outcomes were broken down according to type: infection-related LOS, hospital LOS, and ICU LOS. Consistent with this analysis’s primary hypothesis, heterogeneity was examined primarily for intervention chain types.

For each of the above mortality and LOS types (and for all TTT outcomes), sub-analyses were carried out to determine whether there were differences in the pooled effect based on the category of process improvement implemented. For both LOS and mortality, it was common for authors to report multiple arms (e.g., LOS in the ICU and then LOS in the hospital of the same patients). Where this was the case, hospital LOS and 30-day (or hospital stay alone, if 30-day unavailable) mortality were reported for intervention chain sub-analysis since these were the arms reported most frequently by multi-arm studies. Additionally, sub-analyses were carried out within each outcome type to determine whether the study’s ROB impacted results.

A sensitivity analysis was performed, and formal outliers were dropped. Because of the broad definition of what could count as “process improvements to improve blood culture infection detection and treatment,” a wide variation in the structure of different process improvement efforts was observed—made even more variable by the heterogeneity of the processes and structures in place before the process improvement effort. Thus, a sensitivity analysis dropping the formal outliers provided a sense of the effects that most “average” improvement efforts could expect. Outlier cases were defined as follows.

Those for which the upper bound of the 95% confidence interval (CI) of the study is lower than the lower bound of the pooled effect confidence interval (i.e., extreme overperformance of the intervention). Those for which the lower bound of the 95% CI of the study is higher than the upper bound of the pooled effect confidence interval (i.e., extreme underperformance of the intervention) (42). Outlying studies were reported by the outcome and provided a more detailed examination of “overperforming” and “underperforming” studies.

#### Certainty assessment

The GRADE method of determining the strength of the evidence was used (33).

#### Formulation of the recommendations

The recommendations were initially formulated using the BRIDGE-Wiz software (43). The core team met to discuss and finalize the wording of each recommendation and reach a consensus. Recommendations were labeled according to the GRADE methodology, with “the panel recommends” reflecting a “strong” recommendation and “the panel suggests” reflecting a conditional recommendation. Given that most studies evaluating the impact of diagnostic tests on clinical outcomes will use before-after designs that generally score lower on the strength of the evidence, the core team considered several elements, including the number of studies, the certainty of the evidence, and their expertise in deciding on the strength of the evidence for each recommendation. For the few instances where the evidence was insufficient to direct a recommendation, the panel used their experience and judgment to formulate best practice recommendations following GRADE recommendations (33).

## RESULTS

### Included studies

The search strategies identified 18,642 records, with 17,614 unique records following the exclusion of duplicates. Hand searching of eight meta-analyses identified from the database searches retrieved an additional 510 records, of which 482 were unique records. After automatic deduplication in EndNote, 18,124 unique records were retrieved. From those records, 137 articles were reviewed for eligibility, of which 51 were included in the final analysis after applying inclusion and exclusion criteria (Fig. 1). The narrative summaries and characteristics of the included studies are listed in Table S1.

### Risk of bias analysis summary

**Fig 2:**
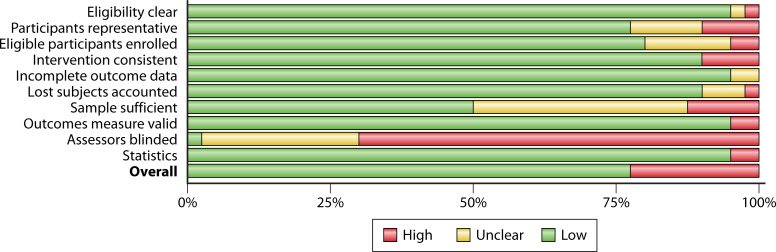
Risk of bias summary plot for before-after designs.

**Fig 3:**
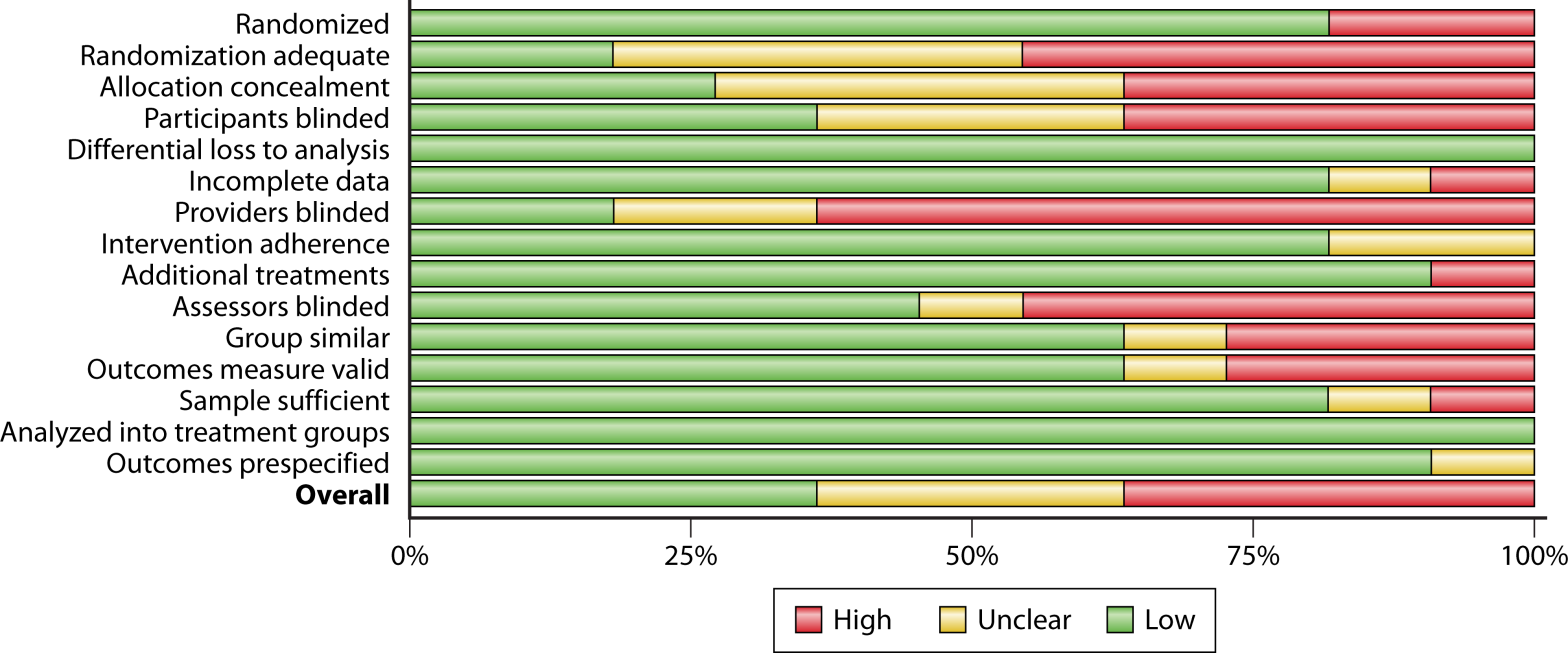
Risk of bias summary plot for controlled study designs.

ROB summary plots for the different classes of study designs used in this analysis are presented in Fig. 2 and 3. Risk of bias criteria for individual studies are presented in the methodological supplement (Fig. S2 to S4). Most studies included in this analysis used before-after designs (pre-implementation/post-implementation). Typically, these studies relied on administrative data and utilized census sampling. The preference for this type of design is unsurprising given the nature of clinic, hospital, or system improvement efforts where expense and training can be extensive (e.g., the cost to purchase PCR device(s) for organism identification and time to implement new procedures across the organization). Using administratively collected data and census sampling (i.e., all patients or samples within a particular time frame) decreased bias. Three limitations were most frequent with before-after designs, two of which were considered serious (Fig. 2).

Process measures were only occasionally reported, so whether interventions were consistent across time and organizational units was sometimes unclear. Additionally, if data were gathered for short periods of time or inclusion criteria were very restrictive (limited to a particular organism), there was some question about whether the sample was sufficient to be confident in the reported effects. Blinding was rarely reported—though, again, this is unsurprising given that it would be exceedingly difficult to blind clinicians to an organization’s improvement effort. Thus, blinding was not considered a serious risk since knowledge and clinician buy-in are crucial for successful process improvement efforts (44). Only one cohort design study was included in this analysis (45). The ROB was considered high due to possible data loss, potentially insufficient sample for the analysis, and lack of confounder adjustment. Controlled studies suffered from many serious ROB concerns. Most serious among these were the adequacy of randomization and lack of blinding (Fig. 3). Additional concerns included whether comparison arms were similar enough to prevent bias (e.g., if different procedures were performed in different hospital units).

### Heterogeneity and sensitivity analysis summary

The pooled effect sizes, observed when rapid diagnostic tests for bloodstream infections were used, are presented in Table 1, sub-stratified by outcome, outcome type, and intervention chain type. For TTT, there was a significant (*P* < 0.001) decrease in the time to targeted treatment (*n* = 34) with an MD of −18.44 hours (95% CI: 25.76, –11.12) across all intervention chain types. Whether the intervention brought about a statistically significant improvement in LOS and mortality depended on the outcome and intervention chain types. For example, five studies used comprehensive intervention chains for infection-related LOS, with a mean reduction of −1.84 days (95% CI: −2.80, –0.88), *P* < 0.001. For comprehensive 30-day mortality overall and for those with a comprehensive intervention chain, the risk ratio (RR) for death was 0.73 (indicating the intervention was 27% protective for the outcome of death) and 0.64 (36% protective), respectively, *P* = 0.19 and 0.003. Unsurprisingly, given the variation in the types of process improvements that could be implemented, heterogeneity was substantial across outcomes; therefore, a sensitivity analysis was performed, excluding outlying studies (Table 2).

**TABLE 1:** Pooled effect sizes broken down by outcome, outcome type, and intervention chain type

	*N* studies	MD^*a*^	MD LCI	MD UCI	I^2^^*b*^	I^2^ LCI	I^2^ UCI	*P*-value
Time to targeted treatment (hours)								
All studies	34	−18.44	−25.76	−11.12	96.9	96.2	97.3	<0.001
Intervention chain types								0.006
Comprehensive^*a*^^*,^c^*^	22	−25.43	−32.79	−18.08	94.5	92.8	95.8	<0.001
Test focused	12	−4.40	−18.63	−9.83	92.6	89.0	95.1	0.545
Length of stay (days)								
All hospitalization	34	−1.28	−2.87	0.30	85.1	80.1	88.8	0.112
Intervention chain types								0.252
Comprehensive	21	−2.00	−4.06	0.05	69.5	52.3	80.5	0.056
Test focused	13	−0.14	−2.58	2.30	87.3	80.0	91.9	0.912
ICU	13	−1.69	−3.66	0.27	97.4	96.5	98.0	0.092
Intervention chain types								0.910
Comprehensive	9	−1.76	−4.73	1.22	79.1	60.7	88.8	0.248
Test focused	4	−1.99	−4.67	0.69	94.8	89.8	97.4	0.146
Infection-related	6	−1.29	−2.87	0.28	65.1	21.5	84.5	0.107
Intervention chain types								<0.001
Comprehensive	5	−1.84	−2.80	−0.88	0.0	0.0	79.2	<0.001
Test focused	1^*d*^	–^*f*^	–	–	–	–	–	–
Mortality (RR^*e*^)								

	*N* Studies	RR	RR LCI	RR UCI	I^2^	I^2^ LCI	I^2^ UCI	*P*-value
Non-specific mortality	7	0.75	0.43	1.31	65.7	23.0	84.7	0.315
Intervention chain types								0.181
Comprehensive	4	1.14	0.84	1.56	0.0	0.0	84.7	0.801
Test focused	3	0.37	0.12	1.13	63.6	0.0	89.6	0.245
30-Day	15	0.73	0.56	0.95	35.9	0.0	65.4	0.019
Intervention chain types								0.228
Comprehensive	9	0.64	0.47	0.86	16.3	0.0	58.4	0.003
Test focused	6	0.88	0.57	1.39	48.8	0.0	79.9	0.605
Hospital mortality	17	0.90	0.76	1.06	0.0	0.0	51.1	0.208
Intervention chain types								0.631
Comprehensive	13	0.87	0.72	1.05	0.0	0.0	57.7	0.084
Test focused	4	1.02	0.67	1.23	0.0	0.0	84.7	0.932
Infection-related mortality	1^*d*^	–	–	–	–	–	–	–

^*a*^ MD, the mean difference (in hours or days); LCI, lower 95% CI; UCI, upper 95% CI.

^*b*^ The I^2^ statistic describes the percentage of variation and inconsistency across study results due to heterogeneity rather than chance. When interpreting I^2^, 0% to 40% might not be important, 30% to 60% may represent moderate heterogeneity, 50% to 90% may represent substantial heterogeneity, and 75% to 100% represents considerable heterogeneity.

^*c*^ Comprehensive: defines the comprehensive intervention chain, including improvements focusing on pre-analytic procedures, organism‘s susceptibility identification, and improved communication strategies. In contrast, Test focused defines improvement strategies focusing only on faster or more accurate testing procedures.

^*d*^ Pooled effect sizes could not be calculated on subgroups with *n* < 2.

^*e*^ RR, the relative risk (or risk ratio), is the ratio of the probability of an outcome in an exposed group (post-intervention or experimental group) to the probability of an outcome in an unexposed group (pre-intervention or control group).

^*f*^ –, no result available.

**TABLE 2:** Sensitivity analysis of effect sizes with outlier studies dropped

	*N* studies	*N* subjects	MD^*a*^	MD LCI	MD UCI	I^2^^*b*^	I^2^ LCI	I^2^ UCI	*P*-value
Time to targeted treatment (hours)	19	6,707	−18.89	−23.36	−14.41	86.8	80.8	90.9	<0.0001
Length of stay (days)									
Hospitalization	28	6,883	−1.45	−2.36	−0.56	39.1	3.9	61.3	0.002
ICU	10	2,843	−0.44	−1.04	0.17	16.7	0	57.8	0.156
Infection-related^*c*^	–^*e*^	–	–	–	–	–	–	–	–

Mortality (RR)	*N* Studies	*N* Subjects	RR	RR LCI	RR UCI	I^2^	I^2^ LCI	I^2^ UCI	*P*-value
Non-specific mortality	6	1,533	1.11	0.83	1.50	0	0	74.6	0.476
30-Day^*d*^	–	–	–	–	–	–	–	–	–
Hospital mortality^*d*^	–	–	–	–	–	–	–	–	–
Infection-related^*d*^ mortality^*d*^	–	–	–	–	–	–	–	–	–

^*a*^ MD, the mean difference (in hours or days); LCI: lower 95% CI; UCI: upper 95% CI.

^*b*^ The I^2^ statistic describes the percentage of variation and inconsistency across study results due to heterogeneity rather than chance.

^*c*^ RR, the relative risk (or risk ratio), is the ratio of the probability of an outcome in an exposed group (post-intervention or experimental group) to the probability of an outcome in an unexposed group (pre-intervention or control group).

^*d*^ Pooled effect sizes could not be calculated on subgroups with *n* < 2.

^*e*^ –, no data exist.

Table 2 provides the effect sizes for the different outcomes when outliers were dropped from the meta-analysis. With outliers excluded, the effect of TTT (*n* = 19, describing 6,707 subjects) increased slightly to an MD of −18.89 hours (95% CI: −23.36, –14.41), and the significance increased 10-fold, *P* < 0.0001, indicating low-performing outliers. The effect for LOS hospitalization (*n* = 28, 6883 subjects) improved from an MD of −1.28 days to −1.45 days (95% CI: −2.36, –0.56) and was statistically significant, *P* = 0.002. When outliers were dropped, the effect size for ICU LOS was not improved, and infection-related LOS could not be calculated. Non-specific mortality increased slightly, but the effect was non-significant. Results for each outcome are presented in additional detail under the summary of the evidence for each recommendation.

## FULL SUMMARY OF RECOMMENDATIONS AND SUPPORTING EVIDENCE

The summary of findings for each study outcome and corresponding outcome sub-analyses, including the evidence profile and certainty of the evidence, are listed in Table 3 and discussed in detail below. Related pooled effect sizes are listed in Table 1.

**TABLE 3:** Evidence profile on the impact of BSI rapid diagnostic tests on clinical outcomes of hospitalized patients

Certainty assessment							No. of patients		Effect		Certainty^*h*^	Importance
No. of studies	Study design	Risk of bias	Inconsistency	Indirectness	Imprecision	Other considerations	Lab process improvement strategies	Standard practice	Relative (95% CI)^*j*^	Absolute (95% CI)		
Time to targeted treatment: observational studies												
31	Observational studies	Not serious^*a*^	Not serious	Not serious	Not serious^*b*^	Publication bias is strongly suspected dose-response gradient^*c*^	3,146	3,749	–^*i*^	MD 16.22 hours lower (23.54 lower to 8.9 lower)	⨁⨁◯◯ Low	Critical
Time to targeted treatment: controlled trials												
7	Controlled trials	Serious^*d*^^,^^*e*^	Not serious	Not serious	Not serious	Publication bias is strongly suspected dose-response gradient^c^	452	459	–	MD 26.14 hours lower (41.58 lower to 10.71 lower)	⨁⨁⨁◯ Moderate	Critical
Hospital LOS: observational studies												
33	Observational studies	Not serious	Not serious	Not serious	Not serious	Dose-response gradient	3,229	4,116	–	MD 2.23 days lower (3.58 lower to 0.88 lower)	⨁⨁⨁◯ Moderate	Critical
Hospital LOS: controlled trials												
8	Controlled trials	Serious^*d*^^,^^*e*^	Serious^*f*^	Not serious	Serious^*g*^	Dose-response gradient	845	844	–	MD 0.01 days higher (3.71 lower to 3.72 higher)	⨁⨁◯◯ Low	Critical
ICU LOS: observational studies												
11	Observational studies	Not serious	Not serious	Not serious	Serious^*g*^	Dose-response gradient	1,084	1,343	–	MD 1.97 days lower (4.18 lower to 0.24 higher)	⨁⨁◯◯ Low	Critical
ICU LOS: controlled trials												
4	Controlled trials	Serious^*d*^^,^^*e*^	Not serious	Not serious	Serious^*g*^	Dose-response gradient	512	503	–	MD 1.41 days lower (3.92 lower to 1.09 higher)	⨁⨁⨁◯ Moderate	Critical
Infection-related LOS: observational studies												
8	Observational studies	Not serious	Not serious	Not serious	Not serious	Dose-response gradient	764	1,060	–	MD 2.13 days lower (3.91 lower to 0.35 lower)	⨁⨁⨁◯ Moderate	Critical
Non-specific mortality: observational studies												
8	Observational studies	Not serious	Not serious	Not serious	Serious^*g*^	None	100/658 (15.2%)	120/818 (14.7%)	**RR 1.05** (0.83 to 1.33)	7 more per 1,000 (from 25 fewer to 48 more)	⨁◯◯◯ Very low	Critical
Non-specific mortality: controlled trials												
2	Controlled trials	Serious^*d*^^,^^*e*^	Not serious	Not serious	Not serious	Strong association	9/259 (3.5%)	38/259 (14.7%)	**RR 0.29** (0.09 to 0.95)	104 fewer per 1,000 (from 134 fewer to 7 fewer)	⨁⨁⨁⨁ High	Critical
30-Day mortality: observational studies												
14	Observational studies	Not serious	Not serious	Not serious	Not serious	None	165/1,654 (10.0%)	287/1,953 (14.7%)	**RR 0.65** (0.52 to 0.82)	51 fewer per 1,000 (from 71 fewer to 26 fewer)	⨁⨁◯◯ Low	Critical
30-Day mortality: controlled trials												
4	Controlled trials	Serious^*d*^^,^^*e*^	Not serious	Not serious	Serious^*g*^	None	52/468 (11.1%)	53/469 (11.3%)	**RR 1.00** (0.70 to 1.43)	0 fewer per 1,000 (from 34 fewer to 49 more)	⨁⨁◯◯ Low	Critical
Hospital mortality: observational studies												
17	Observational studies	Not serious	Not serious	Not serious	Serious^*g*^	Dose-response gradient	212/1,804 (11.8%)	319/2,283 (14.0%)	**RR 0.88** (0.78 to 1.05)	17 fewer per 1,000 (from 31 fewer to 7 more)	⨁⨁◯◯ Low	Critical
Hospital mortality: controlled trials												
4	Controlled trials	Serious^*d*^^,^^*e*^	Not serious	Not serious	Serious^*g*^	None	43/267 (16.1%)	63/277 (22.7%)	**RR 0.74** (0.53 to 1.03)	59 fewer per 1,000 (from 107 fewer to 7 more)	⨁⨁◯◯ Low	Critical
Infection-related mortality: observational studies												
4	Observational studies	Not serious	Not serious	Not serious	Serious^*g*^	None	44/412 (10.7%)	54/380 (14.2%)	**RR 0.69** (0.42 to 1.13)	44 fewer per 1,000 (from 82 fewer to 18 more)	⨁◯◯◯ Very low	Critical

^*a*^ Clinical policy changes or external influences could affect the effect size.

^*b*^ Some variation in error depending on the studies. Only one study with serious imprecision.

^*c*^ Eggers: bias: −2.99, *P* = 0.025.

^*d*^ Adequate blinding was a concern for most studies.

^*e*^ Adequate randomization and allocation concealment were concerns for most studies.

^*f*^ Point estimates vary from clinically large positive effects to clinically large negative effects. Heterogeneity is high and statistically significant.

^*g*^ Confidence Interval for the pooled effect includes zero.

^*h*^ The plus sign inside the circle indicates a positive rating: the more plus signs, the higher the certainty.

^*i*^ – no data are available.

^*j*^ Bold in the p-value column indicates statistical significance.

### Recommendations for the use of rapid diagnostic tests to decrease TTT

### Recommendation 1

To decrease TTT in patients with a positive blood culture, the panel recommends that clinical laboratories implement rapid diagnostics (Evidence quality: Moderate; Recommendation strength: Strong) in combination with a comprehensive plan to actively communicate the actionable test result(s) (Evidence quality: Strong; Recommendation strength: Strong).

#### Remarks

- Most studies evaluating the impact of diagnostic tests on clinical outcomes are retrospective, observational studies that generally score lower on the strength of the evidence scale. However, the panel considered several elements, including the number of studies and the certainty of the evidence, when making the recommendation.
- The panel does NOT recommend using rapid diagnostics tests alone, without active communication, to improve TTT.
- Data were limited for assessing the impact of rapid diagnostics in separate patient populations (e.g., immunocompromised vs immunocompetent hosts).
- There was insufficient evidence to assess the impact of different rapid diagnostic test methods (e.g., NAAT vs MALDI-TOF MS).

### Summary of the evidence

TTT was significantly decreased overall and for comprehensive process improvements (Table 1). There was a significant decrease (*P* < 0.001) in the MD for time to TTT of −18.44 hours (95% CI: −25.76, –11.12) across all intervention chain types (=34). When comparing intervention chain types, the greatest improvement was for comprehensive interventions (*n* = 22), with a reduction in MD of −25.43 hours (95% CI: −32.79, –18.08) (*P*= <0.001). Test-focused improvement strategies (*n* = 12) did not demonstrate statistical significance or clinically meaningful improvement in reducing time to treatment (MD of −4.40 hours [95% CI: −18.63, –9.83], *P* = 0.545).

When outlying studies were excluded, the overall effect on MD TTT improved slightly to −18.89 hours (*P* = <0.0001), indicating low-performing outliers (Table 2).

**Fig 4:**
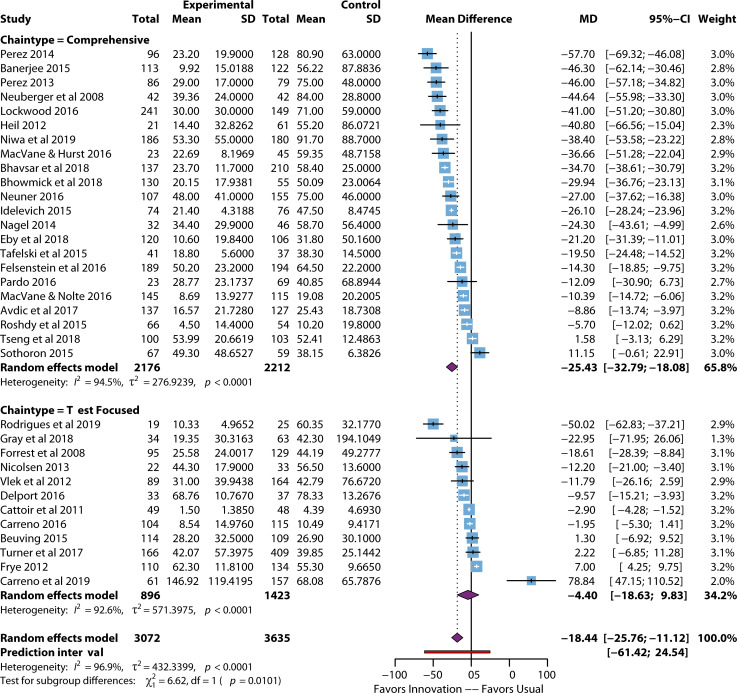
Forest plot of time-to-treatment studies by chain type. References are as follows: Perez et al. (47), Banerjee et al. (48), Perez et al. (49), Neuberger et al. (50), Lockwood et al. (51), Heil et al. (52), Niwa et al. (53), MacVane et al. (54), Bhavsar et al. (55), Bhowmick et al. (56), Neuner et al. (57), Idelevich et al. (58), Nagel et al. (59), Eby et al. (60), Tafelski et al. (61), Felsenstein et al. (62), Pardo et al. (63), MacVane and Nolte (64), Avdic et al. (65), Roshdy et al. (66), Tseng et al. (67), Sothoron et al. (68), Rodrigues et al. (69), Gray et al. (70), Forrest et al. (71), Nicolsen et al. (72), Vlek et al. (73), Delport et al. (74), Cattoir et al. (75), Carreno et al. (76), Beuving et al. (77), Turner et al. (78), Frye et al. (79), Carreno et al. (46).

Figure 4 depicts the left-shifted range of the larger studies (toward shorter TTT time), indicating that the effect range is due more to variation in process improvement practices than to true small study publication bias. Only one large study, Carreno et al. (46), showed a longer TTT with the intervention, notably, a test-only-focused intervention. Some publication bias is possible in the TTT outcome as the funnel plot is asymmetric (Fig. S5) and bias was calculated to be −2.06, *P* = 0.048 (data not shown).

Table S2 also details how studies with a low ROB had a slightly larger and significant subgroup effect. For example, a decrease in TTT (*n* = 28) with an MD reduction at −17.27 hours (95% CI: −25.73, −8.81) compared to the high risk of bias studies (*n* = 8), where the significant subgroup effect was an MD reduction of −19.52 hours (95% CI: −19.52, –9.02). The two studies assessed at moderate ROB had a TTT reduction of −23.85 hours (95% CI: −66.36, –18.65), though this reduction was not statistically significant. While there were small differences in the reduction of TTT based on the overall study ROB analysis, these differences did not reach statistical significance.

There was an unusually high number of outliers (*n* = 15) for the TTT outcome (Table 4). This circumstance is partly due to the natural variation expected in the different process improvement efforts and partly to the relatively small error bands for most studies. The pattern between the overperforming (studies showing an unusually large improvement in TTT) and underperforming (studies showing little or no improvement in TTT) is instructive. While 85.7% (6/7) of overperforming studies used comprehensive improvement chains (linking improvements in test preparation, organism identification, and communication strategies), 75% (6/8) of underperforming studies used only test-focused improvement strategies (e.g., not including efforts to improve the communication of results to clinicians or no active antimicrobial stewardship programs in place). Given these data, the panel strongly recommended that rapid diagnostics be implemented in combination with active communication to ensure optimal impact on TTT.

**TABLE 4:** Characteristics of outlier studies for time to targeted therapy outcomes^*a*^

Studies	Intervention chain type	Comments
Overperforming studies		
Banerjee et al. (48)	Comprehensive	Active antimicrobial stewardship program
Bhavsar et al. (55)	Comprehensive	Active antimicrobial stewardship program
Lockwood et al. (51)	Comprehensive	Active antimicrobial stewardship program
Neuberger et al. (50)	Comprehensive	Positive results were called to the physician
Perez et al. (49)	Comprehensive	Active antimicrobial stewardship program
Perez et al. (47)	Comprehensive	Active antimicrobial stewardship program
Rodrigues et al. (69)	Test focused	Active antimicrobial stewardship program in place before the intervention
Underperforming studies		
Beuving et al. (77)	Test focused	The test only resulted in changes in antibiotics in 12 patients.
Carreno et al. (76)	Test focused	Only septic patients were included. Inconsistent communication
Carreno et al. (46)	Test focused	No power calculations; low numbers
Cattoir et al. (75)	Test focused	No power calculations; small sample size
Frye et al. (79)	Test focused	Only positive intervention was called to the provider by the laboratory
Sothoron et al. (68)	Comprehensive	Most patients in both arms were placed on effective antibiotic(s) shortly after blood collection.
Tseng et al. (67)	Comprehensive	Changes were only made on Gram stain or AST.
Turner et al. (78)	Test focused	No active antimicrobial stewardship program

^*a*^ Additional details are included in the Narrative Summary Table (Table S1).

Heterogeneity (I^2^) for TTT (Table 1) was substantial at 96.9% (95% CI: 96.2, 97.3). Heterogeneity decreased in the outlier analysis (Table 2) to 86.8% (80.8, 90.9) and was still substantial. Heterogeneity in TTT was expected to be large due to the wide variability in global laboratory practices that vary between 24/7/365 and dayshift or no weekend reporting, which could not be subcategorized as specific practices were rarely reported.

### Recommendations for the use of rapid diagnostic tests to decrease LOS

### Recommendation 2

To decrease the hospital LOS in hospitalized patients with a positive blood culture, the panel recommends that clinical laboratories implement rapid diagnostic tests (Evidence quality: Low; Recommendation strength: Strong) in combination with a comprehensive plan to actively communicate the test result(s) (Evidence quality: Moderate; Recommendation strength: Strong).

#### Remarks

- Most studies evaluating the impact of diagnostic tests on clinical outcomes are retrospective, observational studies that generally score lower on the strength of the evidence scale. However, the panel considered several elements, including the number of studies and the certainty of the evidence when making the recommendation.
- The panel considered that the assessment of LOS varied in different hospitals; therefore, given the trend toward lower LOS (impacted by discharge protocols), it decided to weigh the data more heavily and make a strong recommendation.
- The panel does NOT recommend using rapid diagnostics tests alone, without active communications, to decrease hospital LOS.
- There was insufficient evidence to assess the impact of different rapid diagnostic test methods (e.g., NAAT vs MALDI-TOF MS).

### Recommendation 3

To decrease the LOS in the ICU for patients with a positive blood culture, the panel suggests that clinical laboratories implement rapid diagnostic tests (Evidence quality: Moderate; Recommendation strength: Strong) with OR without the healthcare organization implementing a comprehensive plan to actively communicate the actionable test result(s) (Evidence quality: Moderate; Recommendation strength: Weak).

#### Remarks

- Most studies evaluating the impact of diagnostic tests on clinical outcomes are retrospective, observational studies that generally score lower on the strength of the evidence scale. However, the panel considered several elements, including the number of studies and the certainty of the evidence, when making the recommendation.
- The ICU LOS was lower using rapid diagnostics with or without active communication, but the overall number of studies was small. The panel could not recommend for or against using rapid diagnostics to decrease ICU LOS but suggested it as best practice in combination with active communication.
- There was insufficient evidence to assess the impact of different rapid diagnostic test methods (e.g., NAAT vs MALDI-TOF MS).

### Recommendation 4

To decrease the infection-related LOS for patients with a positive blood culture, the panel recommends that clinical laboratories implement rapid diagnostic tests (Evidence quality: Moderate; Recommendation strength: Strong) in combination with a comprehensive plan to actively communicate the test result(s) (Evidence quality: Moderate; Recommendation strength: Strong).

#### Remarks

- Most studies evaluating the impact of diagnostic tests on clinical outcomes are retrospective, observational studies that generally score lower on the strength of the evidence scale. However, the panel considered several elements, including the number of studies and the certainty of the evidence, when making the recommendation.
- The panel does NOT recommend using rapid diagnostics tests alone, although only one study evaluated a “test only” approach to decrease infection-related LOS.

### Summary of the evidence

**Fig 5:**
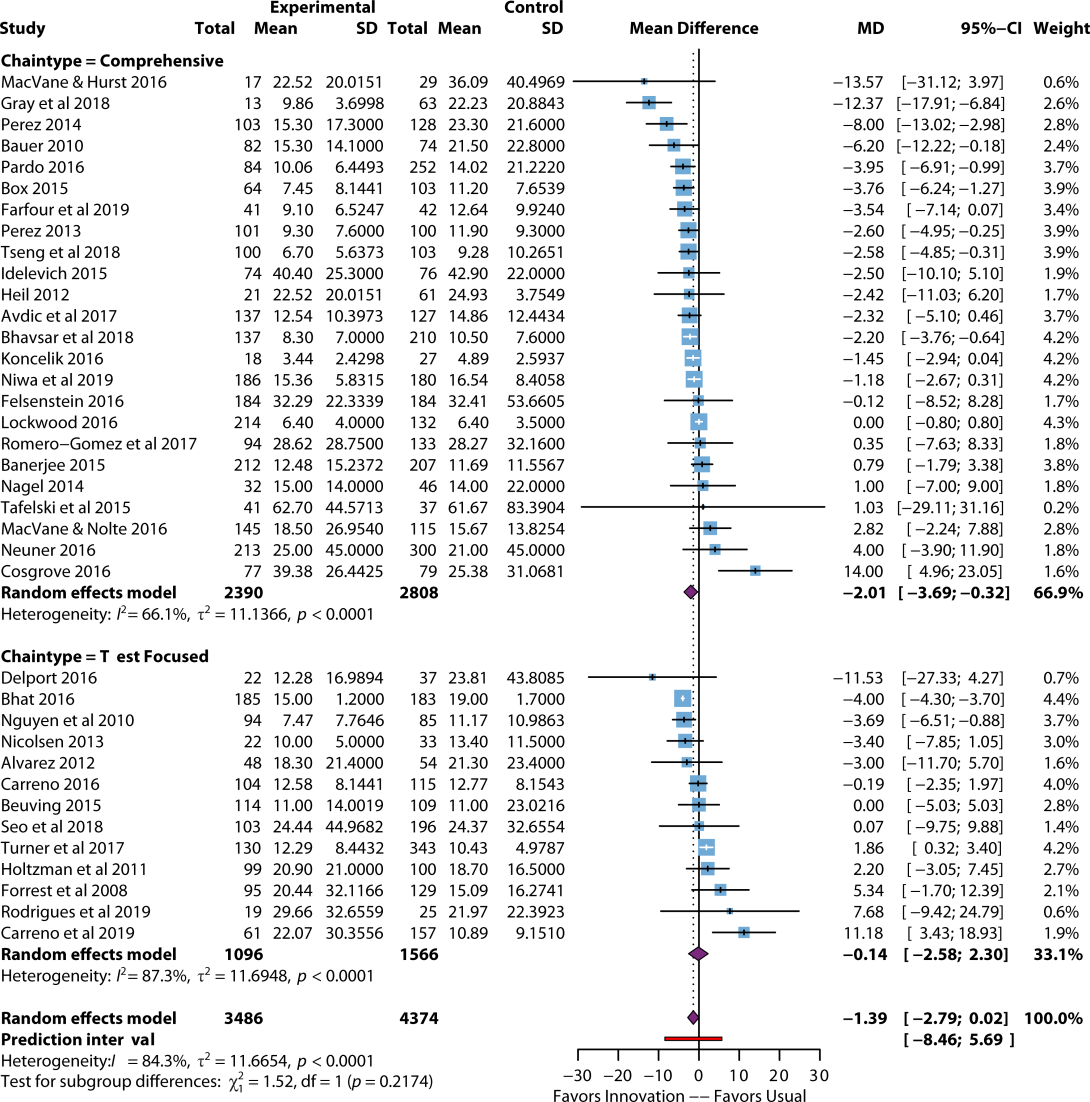
Forest plot of length of stay studies by chain type. References are as follows: MacVane et al. (54), Gray et al. (70), Perez et al. (47), Bauer et al. (81), Pardo et al. (63), Box et al. (82), Farfour et al. (83), Perez et al. (49), Tseng et al. (67), Idelevich et al. (58), Heil et al. (52), Avdic et al. (65), Bhavsar et al. (55), Koncelik et al. (84), Niwa et al. (53), Felsenstein et al. (62), Lockwood et al. (51), Romero-Gomez et al. (85), Banerjee et al. (48), Nagel et al. (59), Tafelski et al. (61), MacVane and Nolte (64), Neuner et al. (57), Cosgrove et al. (86), Delport et al. (74), Bhat et al. (87), Nguyen et al. (88), Nicolsen et al. (72), Alvarez et al. (89), Carreno et al. (76), Beuving et al. (77), Seo et al. (90), Turner et al. (78), Holtzman et al. (91), Forrest et al. (71), Rodrigues et al. (69), Carreno et al. (46).

The LOS outcomes (*n* = 34), listed in Fig. 5, were divided into hospital, ICU, and infection-related subtypes (Table 1). For all three LOS outcomes, there was a reduction in LOS with the process improvement effort; however, none reached statistical differences, except for infection-related LOS with comprehensive intervention chains (*n* = 5), which reduced LOS by −1.81 days (95% CI: −2.8, –0.88), *P* < 0.001. If assessing LOS trends, with a *P*-value cutoff defined as 0.10, then comprehensive interventions resulted in significant reductions in LOS, specifically hospital LOS (*P* = 0.112), ICU LOS (*P* = 0.0962). While the test-focused interventions showed a 1.69-day decrease in ICU LOS, the comparison did not reach statistical significance due primarily to the work reporting increases in ICU LOS (80), thus broadening the confidence intervals of the estimated pooled effect. The authors explained that this unexpected result (i.e., increase in ICU LOS) was likely due to higher “unknown” or “other” sources of infection during the post-implementation period. In addition, the LOS may have been longer for patients who were expected to die but did not.

These considerations point to a fundamental weakness of before-after study designs. An unexpected and unforeseen secular change (e.g., higher infection rates in the post-intervention period, perhaps with nosocomial etiology) may confound the differences between periods. Likewise, since individual risk scores were not reported, assessing the observed-to-expected mortality ratio for each period and controlling for variation were impossible. If the number of infections had increased in the earlier period but not the later period, then the improvements in the ICU LOS could have been large. Without a comparison arm in the same period, it is challenging to establish the true impact of the intervention. The expert panel considered these limitations of the before-after study designs when developing recommendations for LOS.

Hospital LOS and ICU LOS both had outliers that marked an effect change (Tables 2 and 5). With six outliers removed, the MD LOS dropped from −1.28 days to −1.45 days (34.8 hours shorter), *P* = 0.002, *n* = 28 with 6,883 subjects. With three outliers removed for ICU LOS (*n* = 10 with 2,843 subjects), MD LOS dropped by −0.44, which was not statistically significant. No infection-related LOS publications remained when outliers were removed from the analysis.

**TABLE 5:** Characteristics of outlier studies for length of stay outcomes^a^

Studies	Intervention chain type	Comments
Hospital LOS		
Overperforming studies		
Bhat et al. (87)	Test-focused	This was a controlled interventional study in a pediatric patient population using a laboratory-developed test that performed significantly better than the conventional microbiology methods.
Rivard et al. (92)	Comprehensive	The molecular result triggered a real-time electronic message to the antimicrobial stewardship team.
Underperforming studies		
Carreno et al. (46)	Comprehensive	The antimicrobial stewardship team received reports of molecular results twice daily (8 a.m. and 1 p.m.). They saw improved clinical response times but no LOS, TTT, or mortality change. The authors believe the intervention group was more complex than the comparator group.
Cosgrove et al. (86)	Comprehensive	This was a controlled interventional study. The authors shortened the manufacturer’s test protocol by 60 minutes but saw no impact on LOS.
Gray et al. (70)	Comprehensive	The study saw no difference in the LOS, but they did see a significant decrease in the LOS, though in the ICU setting. Testing was performed on *Enterococcus* species only.
Turner et al. (78)	Test-focused	In the absence of an antimicrobial stewardship program, the use of molecular testing alone did not impact the LOS. However, the study was not powered enough to evaluate LOS properly.
ICU LOS		
Underperforming studies		
Gray et al. (70)	Comprehensive	The study saw no difference in the LOS, but they did see a significant decrease in the LOS, though in the ICU setting. Testing was performed on *Enterococcus* species only.
Lockwood et al. (51)	Comprehensive	This was a study performed in two community hospitals where the acuity of patients in the ICU is lower, and the source of BSI is likely from genitourinary sources. Hence, the expected impact of the intervention is lower.
Infection-related LOS		
Underperforming studies		
Turner et al. (78)	Test-focused	Without an ASP, molecular testing alone led to an increase in TTT. However, the study did not have enough power to evaluate clinical outcomes such as LOS adequately.

^a^ Additional details are included in the Narrative Summary Table (Table S1).

Heterogeneity (I^2^) for LOS was substantial for all LOS outcomes, except for infection-related LOS with comprehensive intervention chains (Table 1). This is not surprising given the variations in the makeup of the different process improvement interventions and discharge processes. When outliers were removed from the analysis (Table 2)**,** heterogeneity decreased from 85.1% to 39.1% (95% CI: 3.9, 61.3), indicating that the outlying studies may differ substantially from those retained in the outlier analysis. Heterogeneity for ICU LOS also dropped from 94.7% to a low level of 16.7%. Infection-related LOS could not be analyzed when outliers were removed.

There is weak evidence of publication bias across LOS outcomes (Fig. S6) with bias: calculated to be 1.14, *P* = 0.017 (data not shown). A visual inspection of the funnel plot shows reasonable symmetry, with departures from symmetry in the lower section of the figure favoring increased LOS. Thus, it does not appear that publication bias would result in overestimating the pooled effects, demonstrating a decrease in LOS due to BSI improvement interventions.

In Table S2, the lowest ROB studies in subgroup analysis (*n* = 31) showed the largest reduction in LOS with a decrease of –2.16 days (05% CI: −3.60, –0.71). In comparison, studies with high ROB (*n* = 12) showed a significant effect, with a decrease of −2.16 days (95% CI: −4.05, –0.72). Overall, the subgroup analysis effects did not reach statistical significance (*P* = 0.3927).

For hospital LOS, one comprehensive intervention and one test-focused intervention overperformed the bulk of the other studies (Table 5). Bhat (87) was significant in being the only test-focused study to overperform for any LOS outcome, demonstrating a 4-day decrease in LOS (MD: −4.0, 95% CI: −4.30, –3.70). A plausible explanation may be that while the Bhat 2016 process improvement focused on comparing a molecular organism identification with blood culture, both arms of the study included active communication (via phone calls and emails of the results). This condition contrasts with many other test-focused improvement efforts that did not include active communication components (either as part of the existing process or as part of the process improvement effort).

Given the differences in LOS data, the panel strongly recommended that rapid diagnostics be implemented in combination with active communication to ensure optimal impact on hospital LOS and infection-related LOS. With the acuity of patients in the ICU and their LOS likely being multifactorial, the panel only suggested using rapid diagnostics in combination with active communication to improve the ICU LOS. As most laboratories using rapid diagnostics would likely use panels for ICU patients, this recommendation was provided as a best practice recommendation.

### Recommendations for the use of rapid diagnostics to decrease mortality

### Recommendation 5

To decrease the 30-day mortality in patients with a positive blood culture, the panel recommends that clinical laboratories implement rapid diagnostic tests (Evidence quality: Low; Recommendation strength: Moderate) in combination with a comprehensive plan to actively communicate the actionable test result(s) (Evidence quality: Low; Recommendation strength: Moderate).

#### Remarks

- Most studies evaluating the impact of diagnostic tests on clinical outcomes are retrospective, observational studies that generally score lower on the strength of the evidence. However, the panel considered several elements, including the number of studies and the certainty of the evidence, when making the recommendation.
- The overall impact of rapid diagnostics tests on 30-day mortality varied by study type, with a more significant decrease reported in observational studies.
- The panel does NOT recommend using rapid diagnostics tests alone to decrease 30-day mortality.

### Recommendation 6

To decrease non-specific mortality of patients with a positive blood culture, the panel suggests that clinical laboratories implement rapid diagnostic tests (Evidence quality: Low; Recommendation strength: Weak) in combination with a comprehensive plan to actively communicate the actionable test result(s) (Evidence quality: Low; Recommendation strength: Weak).

#### Remarks

- Most studies evaluating the impact of diagnostic tests on clinical outcomes are retrospective, observational studies that generally score lower on the strength of the evidence. However, the panel considered several elements, including the number of studies and the certainty of the evidence, when making the recommendation.
- The overall impact of rapid diagnostics tests on non-specific mortality varied by study types (i.e., observational vs controlled trials). There was a trend toward lower non-specific mortality, but the difference was not statistically significant.
- The panel could not make recommendations for or against using rapid diagnostics with or without active communication to decrease non-specific mortality but suggested using rapid diagnostics in combination with active communication as a best practice recommendation.

### Recommendation 7

To decrease the in-hospital mortality in patients with a positive blood culture, the panel suggests that clinical laboratories implement rapid diagnostic tests (Evidence quality: Low; Recommendation strength: Low) in combination with a comprehensive plan to actively communicate the actionable test result(s) (Evidence quality: Low; Recommendation strength: Low).

#### Remarks

- Most studies evaluating the impact of diagnostic tests on clinical outcomes are retrospective, observational studies that generally score lower on the strength of the evidence. However, the panel considered several elements, including the number of studies and the certainty of the evidence, when making the recommendation.
- There was an overall trend toward lower in-hospital mortality, but the difference was not statistically significant.
- The panel could not make recommendations for or against using rapid diagnostics with or without active communication to decrease hospital mortality but suggested using rapid diagnostics in combination with active communication as a best practice recommendation. Hospital mortality is likely multifactorial, and many studies were not powered well enough to assess the impact of rapid diagnostics alone.

### Recommendation 8

To decrease infection-related mortality in patients with a positive blood culture, the panel suggests that clinical laboratories implement rapid diagnostic tests (Evidence quality: low; Recommendation strength: Very low) in combination with the healthcare organization implementation of a comprehensive plan to communicate the actionable test result(s) (Evidence quality: low; Recommendation strength: Very low).

#### Remarks

- Most studies evaluating the impact of diagnostic tests on clinical outcomes are retrospective, observational studies that generally score lower on the strength of the evidence. However, the panel considered several elements, including the number of studies and the certainty of the evidence, when making the recommendation.
- There was an overall trend toward lower infection-related mortality, but the difference was not statistically significant.
- Only four studies specifically evaluated infection-related mortality. The panel could not make recommendations for or against using rapid diagnostics with or without active communication to decrease hospital mortality but suggested using rapid diagnostics in combination with active communication as a best practice recommendation. The impact of hospital mortality is likely multifactorial, and many published studies were not powered well enough to assess the impact of rapid diagnostics alone.

### Summary of the evidence

**Fig 6:**
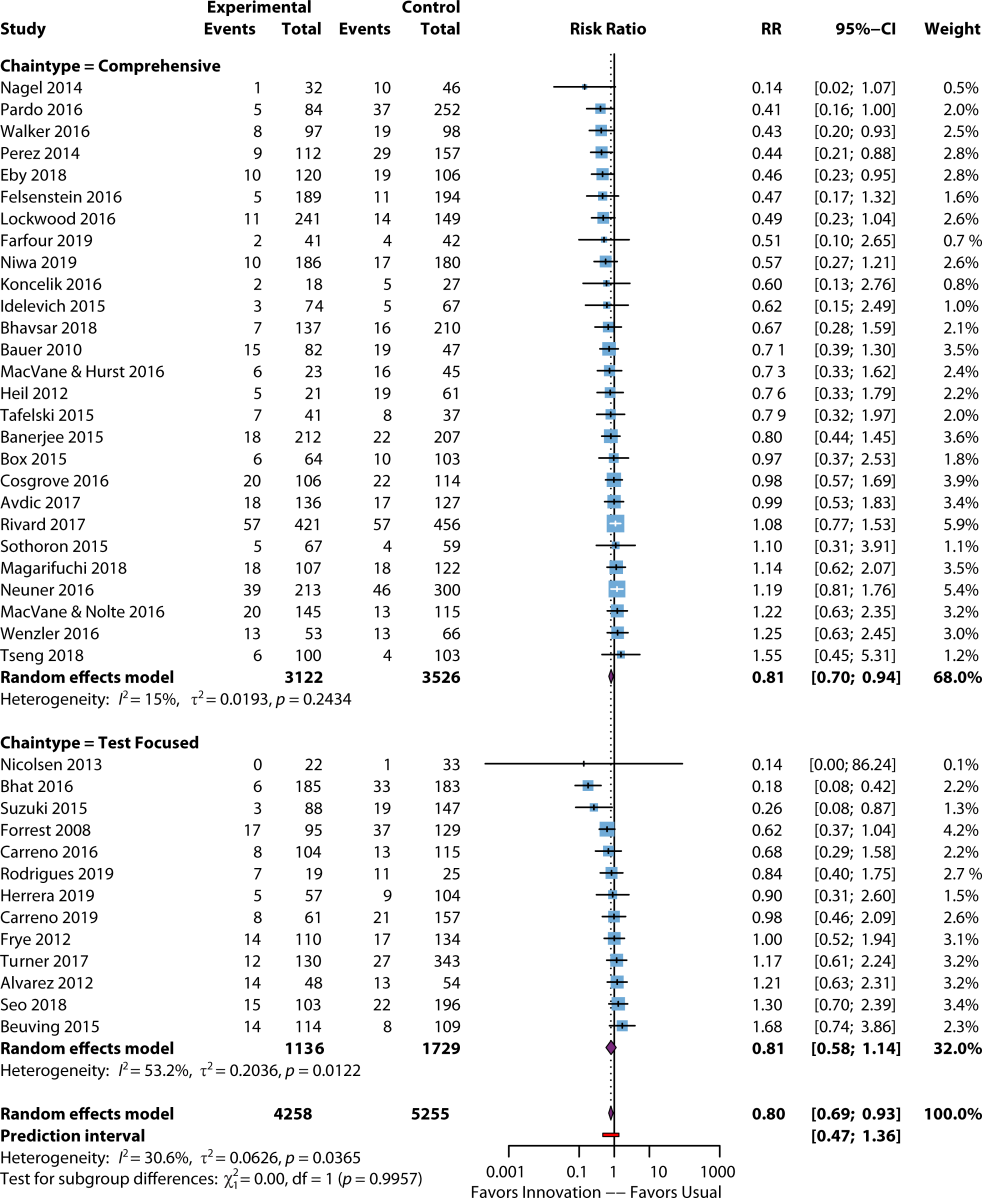
Forest plot of mortality by chain type. References are as follows: Nagel et al. (59), Pardo et al. (63), Walker et al. (93), Perez et al. (47), Eby et al. (60), Felsenstein et al. (62), Lockwood et al. (51), Farfour et al. (83), Niwa et al. (53), Koncelik et al. (84), Idelevich et al. (58), Bhavsar et al. (55), Bauer et al. (81), MacVane et al. (54), Heil et al. (52), Tafelski et al. (61), Banerjee et al. (48), Box et al. (82), Cosgrove et al. (86), Avdic et al. (65), Rivard et al. (92), Sothoron et al. (68), Magarifuchi et al. (94), Neuner et al. (57), MacVane and Nolte (64), Wenzler et al. (95), Tseng et al. (67), Nicolsen et al. (72), Bhat et al. (87), Suzuki et al. (96), Forrest et al. (71), Carreno et al. (76), Rodrigues et al. (69), Herrera et al. (97), Carreno et al. (46), Frye et al. (79), Turner et al. (78), Alvarez et al. (89), Seo et al. (90), Beuving et al. (77).

For mortality, a downstream effect is subject to many factors other than the speed of BSI detection, and the effects of the process improvements were attenuated relative to TTT and LOS. Figure 6 and Table 1 depict the impact of rapid testing on mortality. Only 30-day mortality outcomes (*n* = 15) reached statistical significance, RR = 0.73 [95% CI: 0.73, 0.56], *n* = 15, indicating a 27% lower mortality associated with the process improvement efforts). The mortality improvement was primarily due to comprehensive intervention types, which significantly reduced mortality risk by 36%, with an MD of 0.64 (95% CI: 0.47–0.86, *P* = 0.003); test-focused interventions did not achieve statistical improvement. Hospital mortality risk decreased by 10% (RR 0.90) but was not statistically significant. Only one study by Bhat and colleagues (87) showed a significant decrease in the intervention group (*P* < 0.001), (Fig. 6). It is worth noting that the intervention in this study was a multiplexed laboratory-developed test.

There is no evidence of significant publication bias across the types of mortality outcomes (Fig. S7) with bias = −10.84, *P* = 0.634 (data not shown). While there was no effect on mortality among high- and moderate-risk studies, there was a 25% decrease in risk, which was statistically significant for low ROB studies (*n* = 32) with a RR = 0.75 (95% CI: 0.63, 0.88) (Table S2).

Only non-specific mortality had outliers identified, and one of seven studies was removed, raising the RR from 0.75 to 1.11 (95% CI: 0.83–1.50), but doing so did not reach statistical significance (Table S2). This was generally due to the nature of the metric (not associated with infection), lower sample size, and wide error bands of the included studies. The finding is unsurprising given the “downstream” nature of this outcome (where mortality depends not just on the speed and accuracy of diagnosis but on patient acuity, local differences in care, etc.). For non-specific mortality, Bhat (87), a test-focused intervention, was the only outlier and outperformed other studies in this type of outcome, reducing mortality by 82% compared to the pooled reduction of 25%. With the Bhat 2016 study removed, the pooled effect size for non-specific mortality was a non-significant 11% increase in the mortality rate following the process improvement (RR = 1.11, 95% CI: 0.83, 1.50, *P* = 0.476) (Table 2). Heterogeneity within this outcome decreased with the outlier removed from 51.9% to 0% (95% CI: 0.0%, 74.6%), indicating the strong effect that the Bhat 2016 study had on the heterogeneity measure.

Overall, less impact of rapid diagnostic tests was observed on mortality. Given the challenges of associating mortality exclusively with a BSI, the panel only strongly recommended implementing rapid diagnostics in combination with active communication to decrease the 30-day mortality and suggested using rapid diagnostics in combination with active communication to reduce other types of mortality. Most laboratories using rapid diagnostics would likely use them for many high-risk patients, so these recommendations were provided as a best practice recommendation.

## DISCUSSION

### Summary

BSIs are a significant cause of mortality and morbidity. Rapid identification of bloodstream pathogens via molecular methods or mass spectrometry represents laboratory practices aimed to support fast and actionable changes to antimicrobial therapy, either by escalating therapy to target multidrug-resistant organisms or de-escalating antimicrobial coverage to target the drug and microbe combination better and reduce toxicities caused by broad-spectrum antibiotics. ASM partnered with the CDC and Battelle consultants to perform a systematic review and meta-analysis that was intended to document the impact of the rapid identification of bloodstream pathogens on the downstream clinical outcomes for patients with BSIs (1). Although a trend was detected, indicating that there may be some benefit for patients when the rapid identification of genus and species occurred and was reported to providers with authority for tailoring antibiotics, there were not enough manuscripts for the team to create an official recommendation for evidence-based guidelines. The purpose of this study was to refresh the body of knowledge and evaluate the current evidence for the effectiveness of rapid diagnostic practices to decrease the time to targeted therapy and improve outcomes for hospitalized patients with BSIs, including length of stay and mortality.

A panel of experts, including medical microbiologists and experts in systematic literature review, was assembled to formulate the PICO question, review the literature, and provide recommendations for using rapid tests to diagnose BSI and improve patient outcomes. A comprehensive literature search was conducted to identify studies with measurable outcomes. The panel followed a systematic process that included a standardized methodology for rating the certainty of the evidence and strength of recommendations using the GRADE approach. The panel evaluated the literature to answer the question: in patients with BSI, does using rapid diagnostic tests improve outcomes in adult and pediatric patients hospitalized with bloodstream infections? Peer-reviewed literature was available to address three outcomes, including time to targeted therapy, mortality, and length of stay. The search, from January 2011 through April 2019, identified 18,124 unique records. From those records, 137 articles were reviewed for eligibility, of which 51 were included in the final analysis.

In general, the quality of the evidence was low to moderate due to the paucity of controlled, randomized clinical trial studies. However, eight recommendations were made based on evidence derived from a systematic review and meta-analysis of the published literature. As an answer to the PICO question, the expert committee recommended using rapid diagnostic tests combined with active communication to decrease the time to targeted therapy and length of stay (strong recommendation). While the strength of the evidence for the impact of mortality is low, the panel supports using rapid tests to impact these outcomes.

### Applicability/generalizability

The panel recommends using rapid diagnostics in combination with active communication. Active communication can be accomplished by phone calls, traditional pages, or text pages with return receipts with infectious disease providers, pharmacists, or other patient care team members to guide evidence-based antimicrobial therapy and stewardship practices. There was not enough evidence to assess the impact of different rapid diagnostic tests separately. The studies reviewed in this guideline included adult and pediatric patients, but the ability to assess different patient populations was limited. Therefore, results might not be generalizable to all hospitals, including specialized healthcare systems.

### Associated harms

All laboratory tests can produce false-negative or -positive results, possibly leading to inappropriate antimicrobial usage. Other harms of rapid molecular testing may occur when blood culture broths contain DNA from non-viable microbes, which are introduced into the preparation of the blood culture broth. While most molecular manufacturers support a lower limit of detection of microbial DNA well above the signal of low-level DNA contamination, the bottles themselves are not DNA-free. Occasionally, a large bolus of bacterial or fungal DNA enters the manufacturing process and causes false-positive results. A process for comparing the Gram stains of bottles containing viable microbes with the results of rapid molecular testing is warranted (https://asm.org/Guideline/Guidelines-Regarding-Genotypic-False-Detections-Fr). Such comparisons mitigate the impact of false-positive molecular results that may lead to antimicrobial therapy that could be harmful to the patient. Additional associated harms could derive from inadequate oversight of personnel performing moderate complexity testing 24-7-365, or from allowing staff to wait for the molecular test results before performing the Gram stain. The workgroup considers the Gram stain the first actionable test result, followed by the rapid molecular test.

### Additional benefits

Beyond the benefits investigated in this guideline, the use of rapid diagnostics for BSIs may have additional benefits that might be harder to confirm through a standard meta-analysis given that type of data is not always published. First, the FDA categorizes many of the tests in this guideline as moderate-complexity tests, which are then monitored by CMS or one of its deemed agents. As such, tests are designed to both be user-friendly and require minimal hands-on time to run the test, and the skill level necessary to accurately perform and interpret the test allows for the expansion of the technical workforce to non-microbiology technologists, particularly in cases where a Gram stain does not need to be interpreted to perform the test. Given the well-documented critical shortage of medical laboratory professionals, moderate-complexity diagnostic tests that laboratory professionals can perform without specialized microbiology training are a significant added benefit. Second, while there is a historical trend toward centralizing laboratory testing, the availability of rapid tests could provide a simple option to perform the initial testing of positive blood cultures closer to the point of collection and initial incubation of the blood cultures rather than at a remote, central laboratory. This added benefit would further decrease the time to initial results without requiring highly trained microbiologists to be available on-site 24/7/365.

### Feasibility of implementation and practical considerations

The practical considerations for the adoption of the guideline should include a focus on adapting it to local conditions, microbial prevalence, and healthcare infrastructure. Hospital-based laboratory leadership is encouraged to seek input from key stakeholders such as infectious disease providers, ASP team members, DSP team members, critical care and emergency department providers, nursing staff, pharmacy staff, finance, IT, and quality officers to adopt the guidelines locally. Discussion should include the processes by which the actionable data from the rapid results are communicated and to whom. A clear laboratory quality plan should be in place before deployment, encompassing pre-analytic, analytic, and post-analytic plans.

Pre-analytical components are critical. Minimizing skin contaminants in the laboratory will save money for the laboratory by not testing microbes that may not be pathogenic. Monitoring the entire blood culture process is a realistic requirement for optimal deployment since transport delays hinder the optimal time for actionable results.

The Health Information Technology and Electronic Health Record components are also critical for analytic workflow. For example, hardwiring the rapid results into a reflex pathway if the culture is positive and determining when and if repeat testing will occur are important to communicate with stakeholders and providers. Following up with stakeholders when rapid testing is positive, but microbes do not grow on subculture, is also a process that should be in place before deployment. Laboratories should create communication plans for their providers and for modified laboratory procedures for reporting test results when and if the bottles from the manufacturer are shipped with non-viable DNA, which could cause false-positive results.

Monitoring post-analytical actionability will also be critical, as results without action for de-escalation or escalation could amount to wasted costs and laboratory effort. Dashboard alignment of laboratory and pharmacy timelines is an effective way to ensure success. Laboratory requirements that use text- or phone-based pages about actionable results are likely to be a critical step in the overall goals of saving and improving lives and cost savings from earlier hospital discharges.

### Limitations

A few limitations are reflected in the guideline process for BSI rapid diagnostics. First, the analysis did not include sub-stratification between outcomes for NAAT, hybridization, or MALDI-TOF MS rapid methods. The latter provides slower results; therefore, their combination may have reduced the impact of the molecular methods. Secondly, gram-negative vs gram-positive organism substrata are not reported here, and the impact of rapid testing may differ among different Gram categories, genus, or even species. Furthermore, no standard antimicrobial escalation or de-escalation algorithms are represented in the analysis because each study and healthcare system independently defines its formulary and antimicrobial stewardship guidelines. Likewise, there is no substratification between adult and pediatric patients because of the exclusion of pediatric bottles from some FDA approvals and the lack of pediatric-focused publications. The same limitation applies to the use of rapid diagnostics used for anaerobic bottles, which support the growth of microaerophilic microbes but are not FDA-approved for use with rapid diagnostics, even though they often flag positive before the aerobic bottle does. Finally, the guideline does not represent the complexities of polymicrobial BSIs as those infections were either not addressed, excluded, or represented only first positive microbes.

The development of this guideline was initially started following the A-6 method, which was similar to the original guideline published in 2016. However, the ASM leadership decided to align its approach to guideline development with established protocols from the Institute of Medicine’s practice standards and the GRADE approach (32). Hence, direct comparison to the 2016 ASM guideline is limited. Despite the extensive efforts, systematic reviews are not without limitations. The reviews generally seek to answer a narrow and specific question rather than a wide breadth of related topics. Also, conducting a meta-analysis does not guarantee that there will be no bias in the individual studies contributing to the synthesis. Finally, and most importantly, there are still a limited number of high-quality outcome-based studies to inform the systematic reviews and meta-analysis for other pressing quality questions. With more laboratorians aware of and publishing in these categories, the ability of ASM to develop future evidence-based guidelines should improve.

## FUTURE RESEARCH NEEDS

The initial scope of this guideline was meant to be more comprehensive. Following the scoping review (4), the scope was adjusted due to the lack of evidence to investigate other outcomes and diagnostic interventions. Further research is needed to fill these gaps in knowledge. The expert panel developed the following questions to inform future research.

- How do rapid diagnostic methods impact clinical outcomes for pediatric patients?
- Do rapid diagnostics result in decreased laboratory and/or hospital costs?
- Are rapid diagnostics for BSI effective at decreasing antibiotic use and/or duration?
- Do rapid diagnostics for BSI impact the number of infectious disease consults?
- How do rapid diagnostics impact clinical outcomes such as mortality in patients with fungemia?
- How do rapid diagnostics impact operational outcomes such as hospital length of stay and overall hospitalization costs?
- How does rapid diagnostics impact the time to targeted therapy for patients with fungemia?
- What is the utility of rapid diagnostics performed directly on EDTA blood samples?
- When novel, rapid phenotypic tests are deployed, what are the clinical, operational, and financial impacts on patient outcomes? Is there a difference in clinical outcomes among different patient populations?
- What is the utility of host-response marker tests on patient outcomes?
- How rapid is rapid enough to have a maximum impact on patient outcomes?
- What is the impact of 24/7/365 testing in different patient populations?
- Does the impact differ by the specific method of active communication or the robustness of the organization’s ASP or DSP?
- How do delays in blood culture transport to a core laboratory affect clinical, operational, and financial outcomes?
- How will the new FDA (Food and Drug Administration) regulation for laboratory-developed tests impact the use of rapid diagnostic methods on the anaerobic bottle, in which many microaerophilic microbes are detected before the FDA-approved use of the aerobic blood culture bottle?
- How will the new FDA (Food and Drug Administration) regulation for laboratory-developed tests impact the use of rapid diagnostic methods on pediatric bottles?

## CONCLUSIONS

The guideline panel used a methodologically rigorous process to critically appraise available literature on rapid diagnostic tests for identifying bacterial pathogens causing bloodstream infections. While the evidence available has increased since the last guideline document was published, the literature is still limited due to the number of studies with bias and low-quality evidence. However, based on the moderate certainty of the evidence, the ASM panel strongly recommends using rapid diagnostics in combination with active communication by ASPs for blood cultures positive for bacterial pathogens. The ASM panel also suggests using rapid diagnostics to rapidly identify antibiotic resistance markers that can inform more targeted therapy. An evidence gap precluded recommending direct-from-blood samples diagnostics (culture-free) and rapid diagnostics for organisms other than bacteria (e.g., fungemia). The critical components of future diagnostic studies include well-designed studies to increase the strength and quality of the evidence. In the 2016 publication, Buehler et al. (1) provide an appendix that lists relevant study designs, outcomes, and control variables that could be used by those who plan to investigate the impact of rapid diagnostics for bloodstream infections in their local organization. Given the nature of laboratory studies, the design might be limited to before and after studies; however, it can still provide useful information if sufficient details are included in the study and ROB is limited.

## EDITOR'S NOTE

**Ed. Note**: A potential conflict of interest was identified after acceptance of this paper, and the Editor in Chief performed an additional final review of the paper.

## SUPPLEMENTAL MATERIAL

Fig. S1 cmr.00137-24-s0001.tif Algorithm to determine the formula to estimate means and standard deviations.

Fig. S2 cmr.00137-24-s0002.tif Risk of bias by study-before-after design.

Fig. S3 cmr.00137-24-s0003.tif Risk of bias by study - cohort designs.

Fig. S4 cmr.00137-24-s0004.tif Risk of bias by study - controlled study designs.

Fig. S5 cmr.00137-24-s0005.tif Study bias for time-to-targeted treatment outcomes.

Fig. S6 cmr.00137-24-s0006.tif Study bias for length of stay outcomes.

Fig. S7 cmr.00137-24-s0007.tif Study bias for mortality outcomes.

Supplemental Tables 1 and 2 cmr.00137-24-s0008.docx Table S1 (Narrative summary and characteristics of included studies) and Table S2 (Subgroup analysis for risk of bias by outcome).

Supplemental Table and Figure Legends cmr.00137-24-s0009.docx Supplemental Table and Figure Legends.

Text S1 cmr.00137-24-s0010.docx Executive summary and recommendations.

ASM does not own the copyrights to Supplemental Material that may be linked to, or accessed through, an article. The authors have granted ASM a non-exclusive, world-wide license to publish the Supplemental Material files. Please contact the corresponding author directly for reuse.
