# Supplementary figures and images for: The American Society for Microbiology’s evidence-based laboratory medicine practice guidelines for the diagnosis of bloodstream infections using rapid tests: a systematic review and meta-analysis
Source: Clin Microbiol Rev. 2025 Jun 16;38(3):e00137-24. doi: 10.1128/cmr.00137-24 (PMC12424361; doi:10.1128/cmr.00137-24)

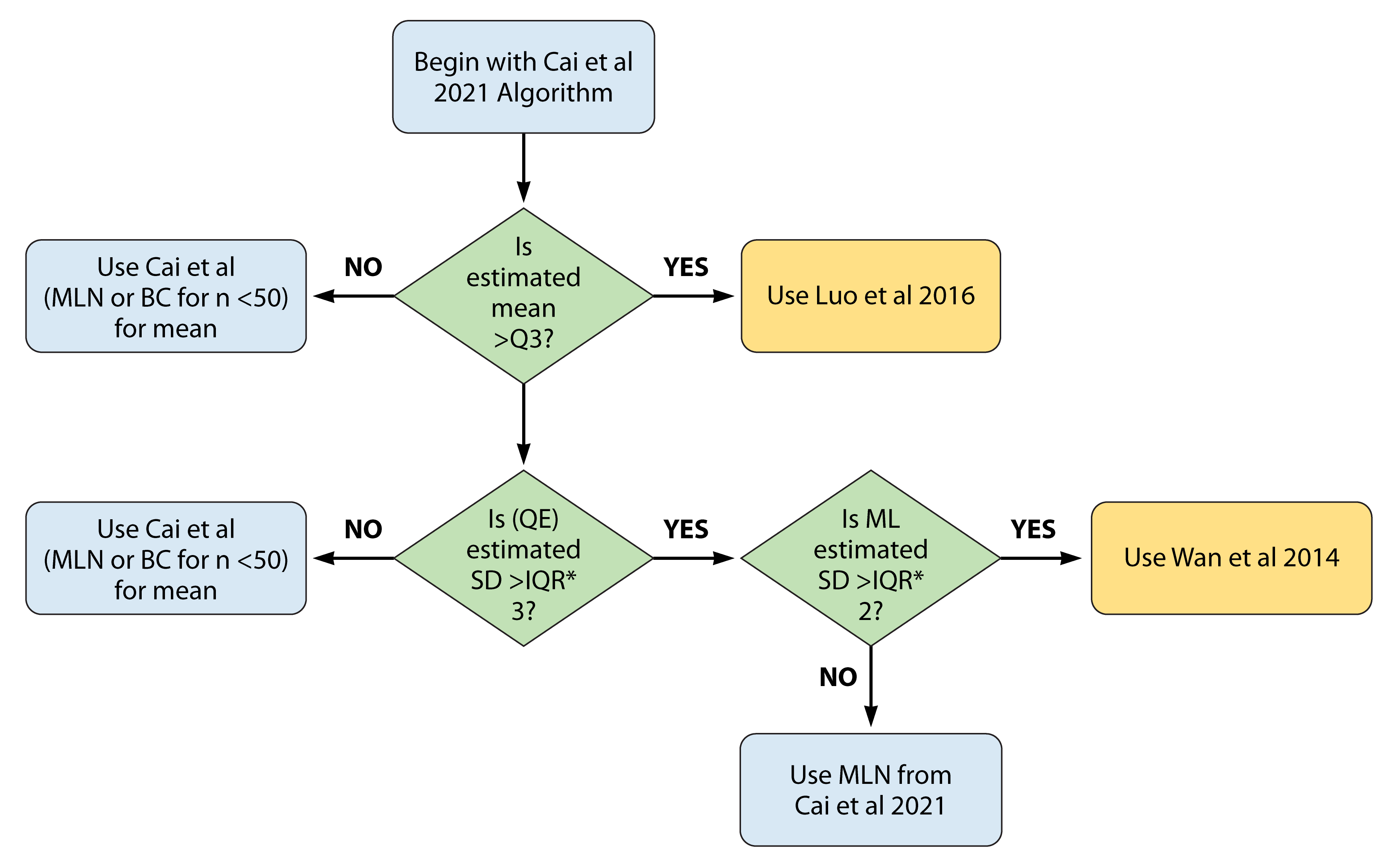

Supplement: Fig. S1 — Algorithm to determine the formula to estimate means and standard deviations. [file cmr.00137-24-s0001.tif]

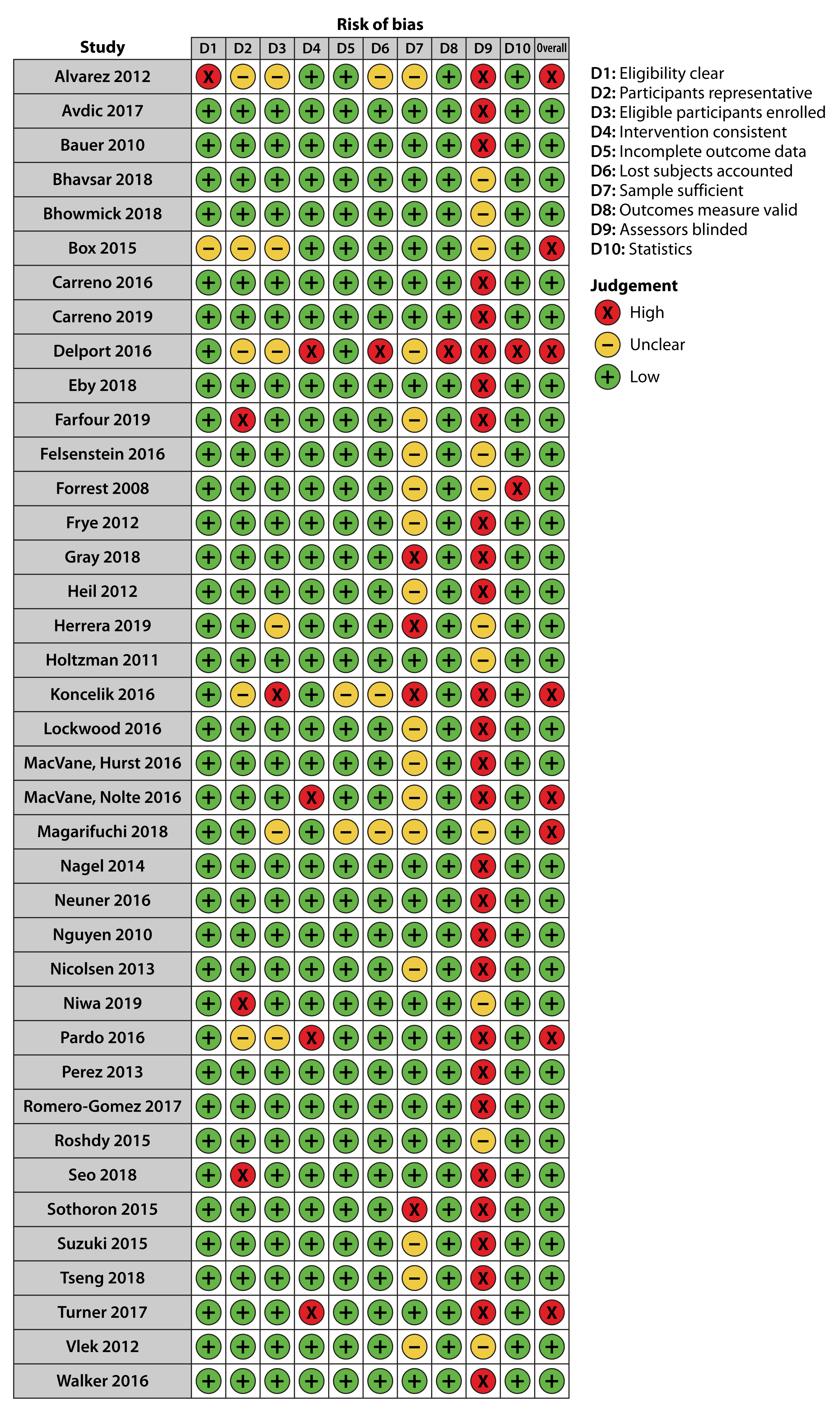

Supplement: Fig. S2 — Risk of bias by study-before-after design. [file cmr.00137-24-s0002.tif]

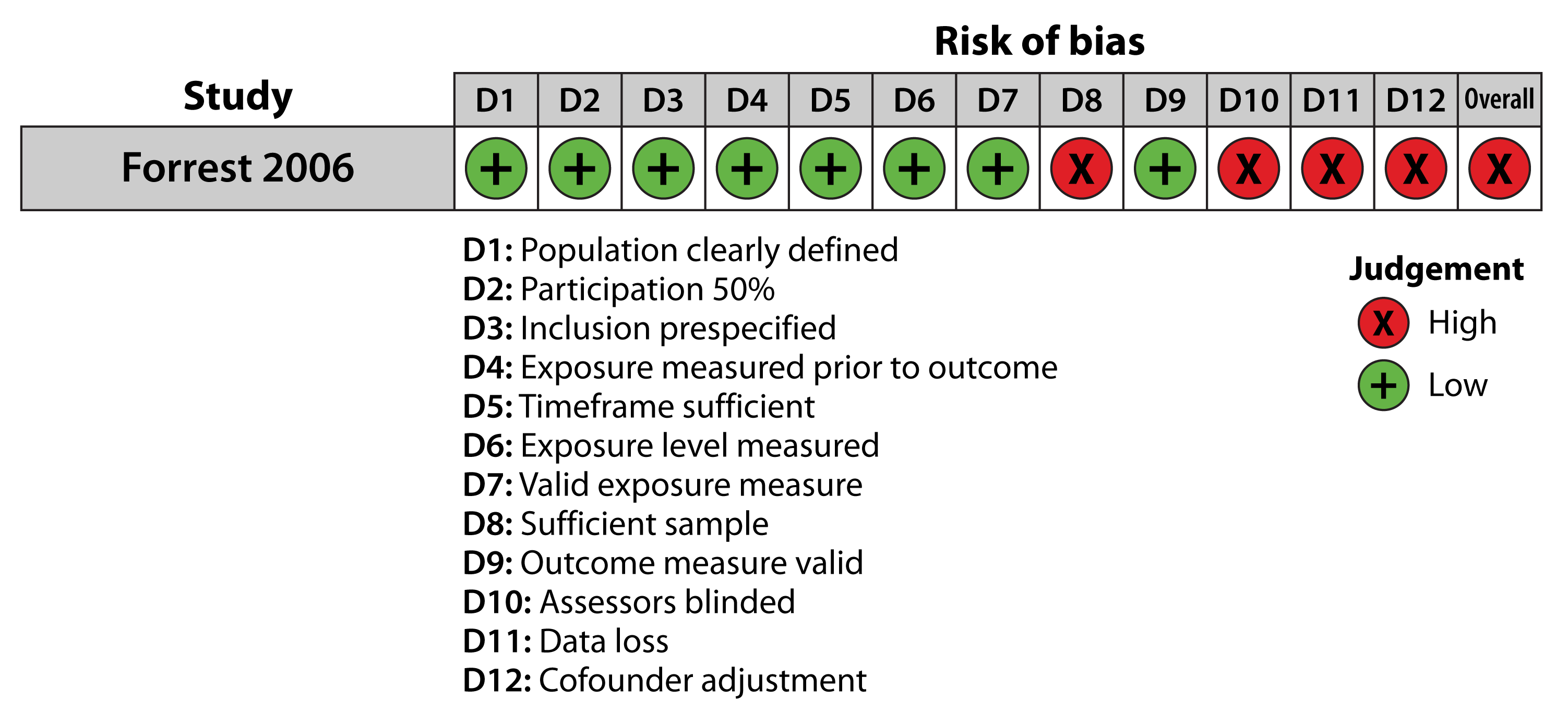

Supplement: Fig. S3 — Risk of bias by study - cohort designs. [file cmr.00137-24-s0003.tif]

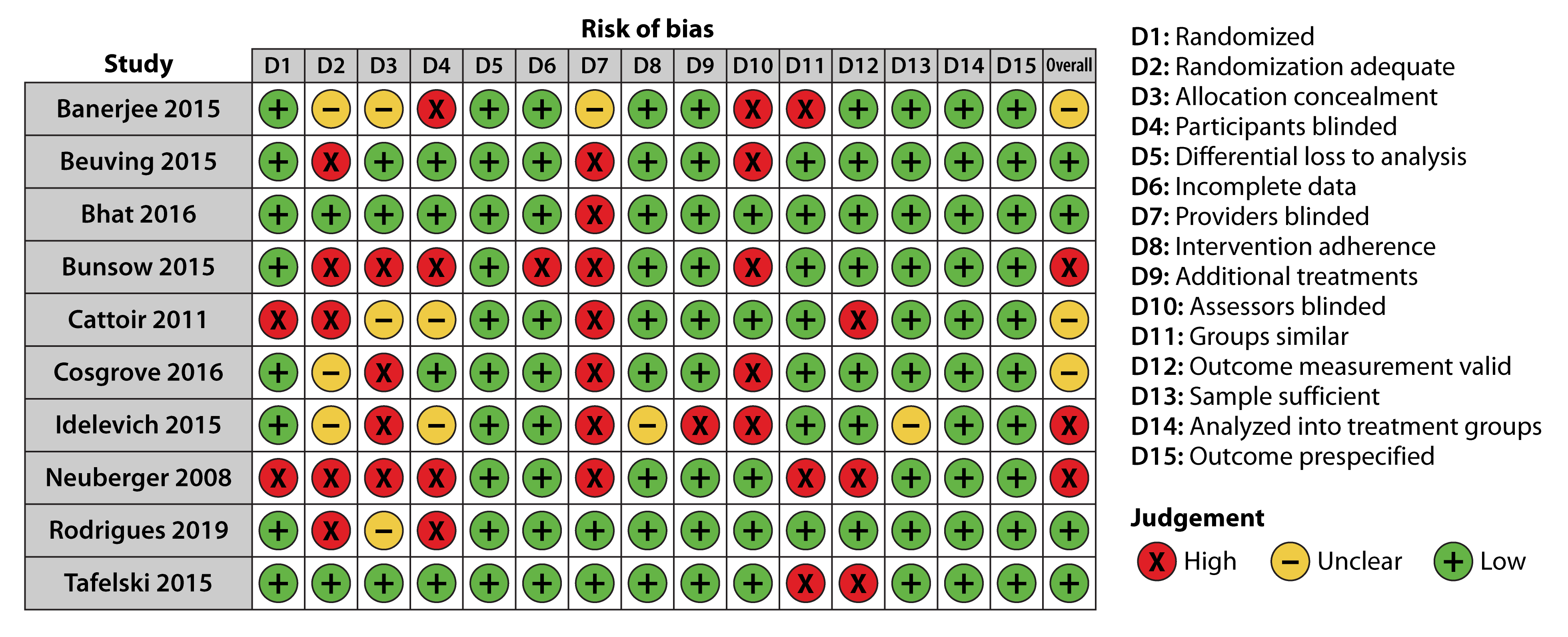

Supplement: Fig. S4 — Risk of bias by study - controlled study designs. [file cmr.00137-24-s0004.tif]

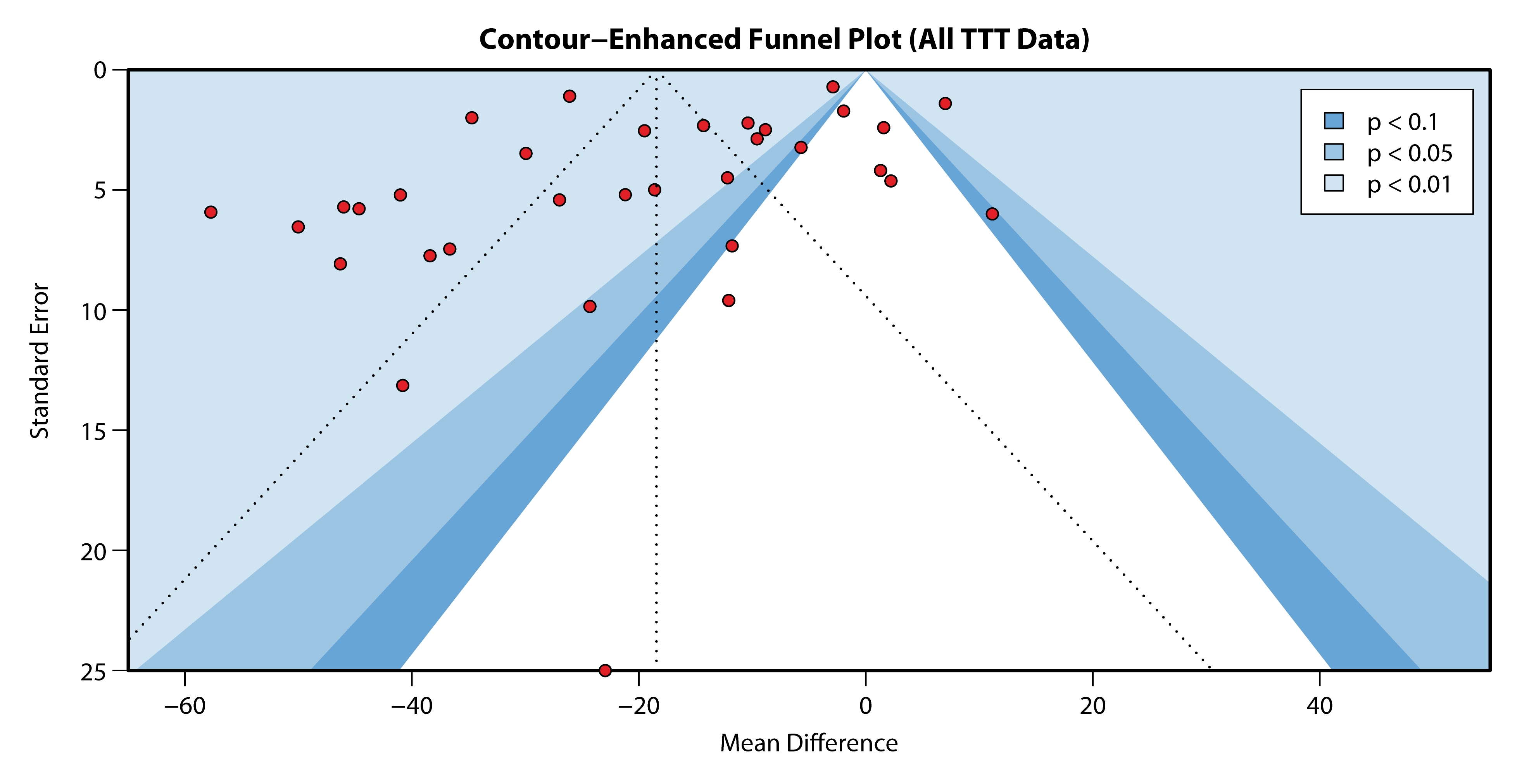

Supplement: Fig. S5 — Study bias for time-to-targeted treatment outcomes. [file cmr.00137-24-s0005.tif]

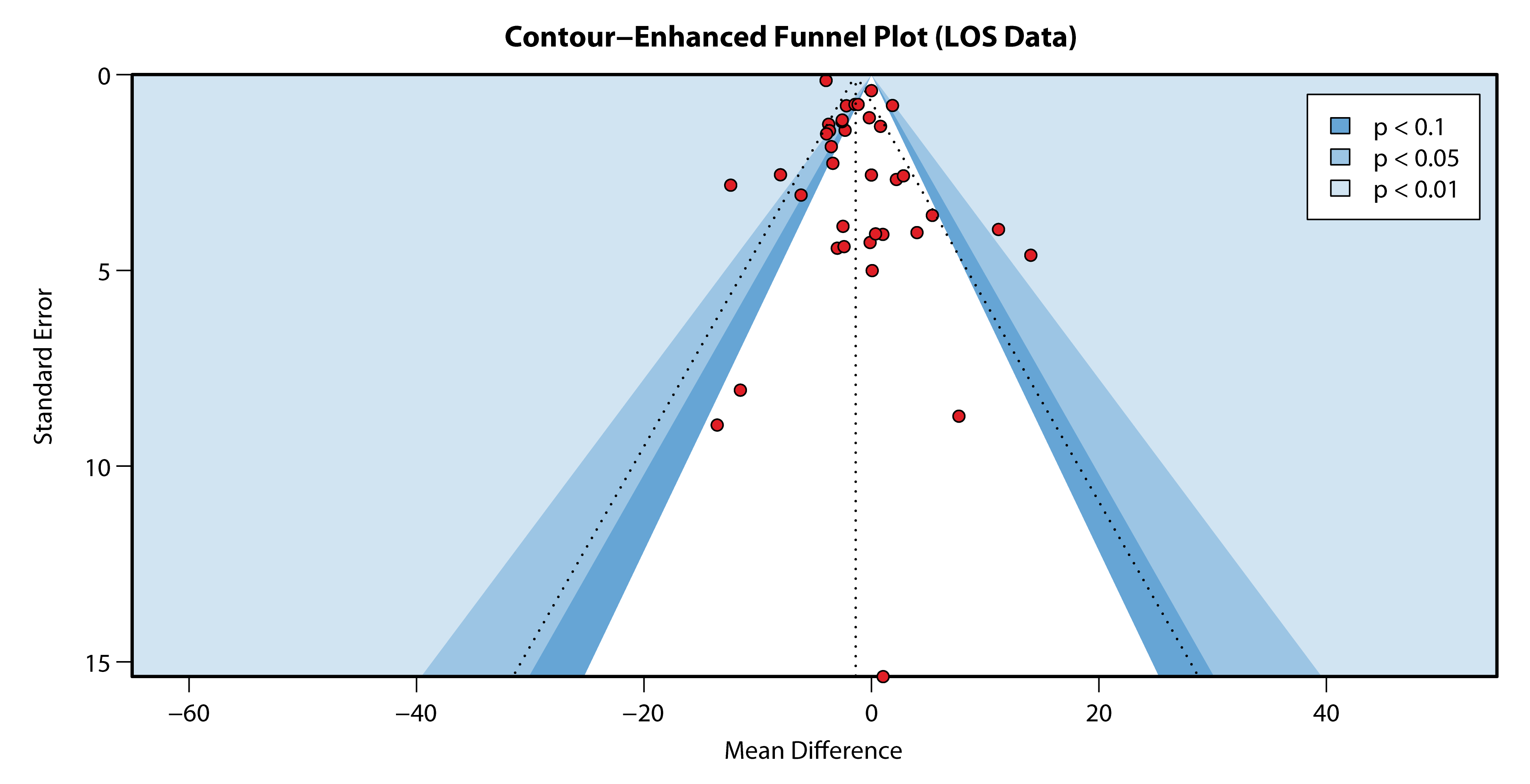

Supplement: Fig. S6 — Study bias for length of stay outcomes. [file cmr.00137-24-s0006.tif]

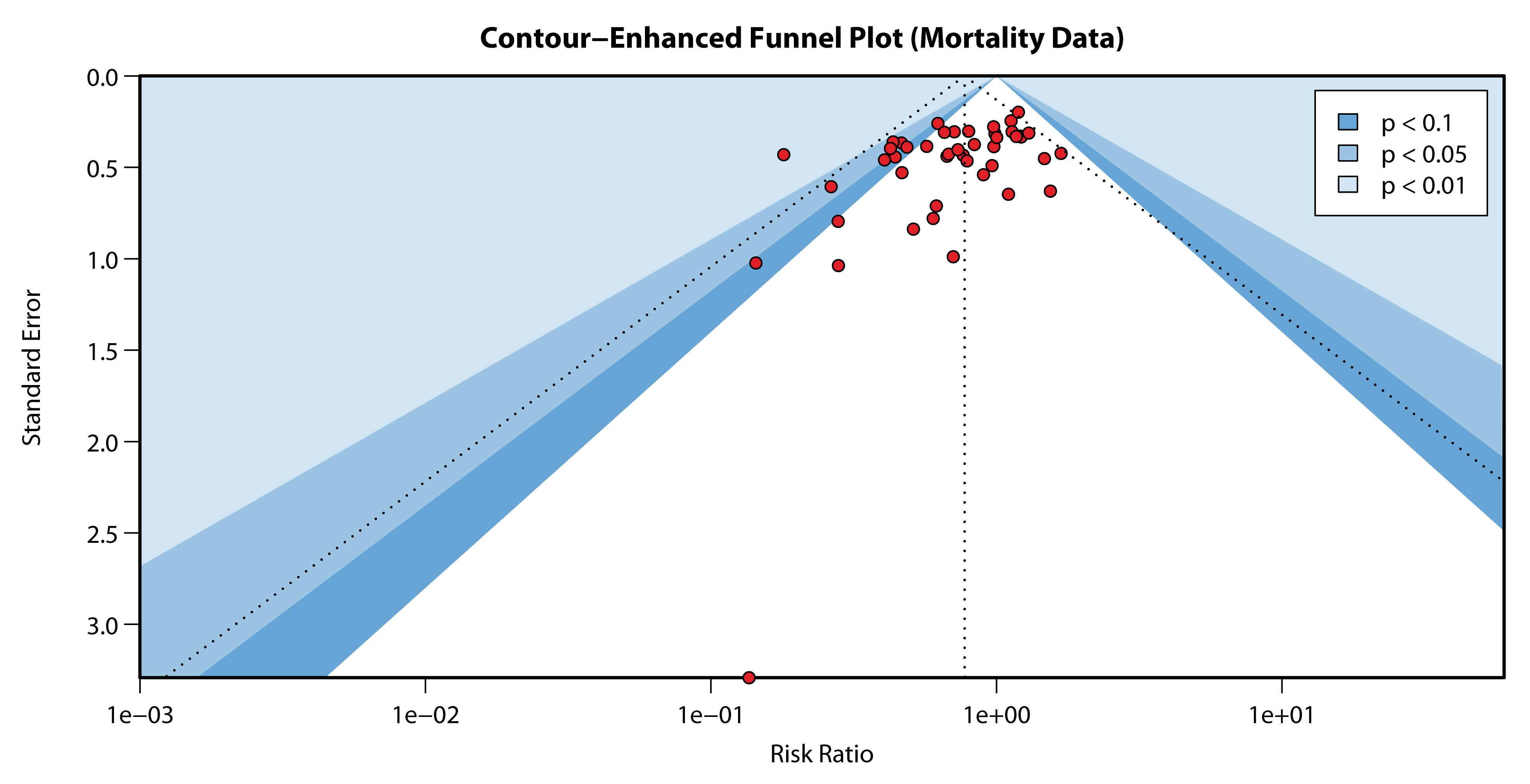

Supplement: Fig. S7 — Study bias for mortality outcomes. [file cmr.00137-24-s0007.tif]
