# Supplementary material for: The American Society for Microbiology’s evidence-based laboratory medicine practice guidelines for the diagnosis of bloodstream infections using rapid tests: a systematic review and meta-analysis
Source: Clin Microbiol Rev. 2025 Jun 16;38(3):e00137-24. doi: 10.1128/cmr.00137-24 (PMC12424361; doi:10.1128/cmr.00137-24)
Supplement: Supplemental Tables 1 and 2 — Table S1 (Narrative summary and characteristics of included studies) and Table S2 (Subgroup analysis for risk of bias by outcome). [file cmr.00137-24-s0008.docx]

**Table S1. Narrative Summary and Characteristics of Included Studies** (see reference list at the end of this document)

| **Author**  **Year** | **Location Year of recruitment** | **Design** | **Type and Number of patients** | **Inter-vention** | **Regulatory status** | **Comparator** | **Measured outcomes** | **Notable Limitations** | **External Funding** |
| --- | --- | --- | --- | --- | --- | --- | --- | --- | --- |
| Alvarez, 2012 | Santiago de Compostela, Spain;  Comparator period: January-June 2006 Intervention period: July-December 2006 | Before-After (Pre-Post) Studies with No Control Group | Adult  Comparator group: 54 patients Intervention group: 48 patients | NAAT | CE-Marked | Conventional Microbiology | **Hospital LOS:** 21.3 pre vs 18.3 days post-intervention, p-value <0.05. **ICU LOS:** 31.0 pre vs 22.9 days post-intervention, p-value <0.05. **Mortality:** 28-day mortality was not different between the two groups (24% pre- vs. 29% post-intervention), and 6-month mortality was not different between the two groups (37% pre- vs. 41.6% post-intervention). | No description of conventional microbiology | No external funding |
| Avdic, 2017 | Baltimore, Maryland, USA;  Comparator period: August 2012-March 2013 Intervention period: March 2013-May 2014 | Before-After (Pre-Post) Studies with No Control Group | Adult  Comparator group: 125 patients  Intervention group 1: 134 patients Intervention group 2: 131 patients | NAAT | FDA-approved | Conventional Microbiology;  PNA FISH | **TTT:** The median TTT was significantly shorter only for MSSA bacteremia in post-intervention periods compared with the baseline period (17 versus 50 h; p-value < 0.001). **LOS:** There was no difference in LOS between the comparator and intervention groups (11 days for both groups, p-value = 0.81). **Mortality:** In-hospital mortality was not different between the three groups (n=17 pre- vs. 17 in the intervention (p-value = 0.86) vs. 18 in post-intervention; p-value = 0.99). | ND | No external funding |
| Banerjee, 2015 | Rochester, Minnesota, USA;  August 2013-March 2014 | Controlled Intervention Study | Adult  Comparator group: 207 patients  Intervention group 1: 198 patients Intervention group 2: 212 patients | NAAT | FDA-approved | MALDI-TOF MS; PBP-2 | TTT: The time from Gram stain to de-escalation was shortest in the intervention with the stewardship arm (21 vs. 34 hrs., p-value < 0.001) as well as time to escalation (5 hrs. in the intervention arm vs. 24 hrs. in the control arm, p-value = 0.04).  LOS: There were no differences in hospital LOS among the three groups (8 days for all three groups, p=0.60) and no differences in ICU LOS (3 days in the control and intervention with stewardship and 2 days in the intervention arm, p-value = 0.9).  Mortality: 30-day mortality was not different between the three groups (10.6% pre- vs. 10.1% in the intervention vs. 8.5% in intervention+stewardship; p-value = 0.74), 30-day attributable mortality was not different between the three groups (3.4% in the control vs 3.5% in the intervention vs 0.9% in the intervention+stewardship, p-value = 0.42). | ND | Government grant & manufacturer/industry funding |
| Bauer, 2010 | Columbus, Ohio, USA;  Comparator period: September-December 2008 Intervention period: March-June 2009 | Before-After (Pre-Post) Studies with No Control Group | Adult  Comparator group: 74 patients Intervention Group: 82 patients | NAAT | FDA-approved | Conventional Microbiology | **TTT:** The TTT was significant in patients with MSSA bacteremia in the post vs pre-intervention arm (2.0 vs 3.6 days, p-value = 0.002). **LOS:** The mean LOS was lower in the comparator vs intervention group (21.5 vs 15.3 days; p-value = 0.07).  **Mortality:** Hospital mortality was not different between the two groups (26% comparator vs 18% intervention, p-value =0.33). | No description of conventional microbiology | Manufacturer/industry funding |
| Beuving, 2015 | Maastricht, Netherlands;  October 2009-May 2011 | Controlled Intervention Study | Adult  Comparator group: 121 patients Intervention group: 129 patients | NAAT | LDT | Conventional Microbiology | **TTT:** The mean TTT was statistically significantly lower in the comparator group than in the intervention group (42.3 h vs 65.9 h, p-value <0.001). **LOS:** There were no differences in LOS between the two groups (11 days for both groups, p-value = 0.820. **Mortality:** 30-day in-hospital mortality was not different between the two groups (12% in the intervention arm vs 7.3% in the comparator group, p-value = 0.216). | ND | Foundation grant |
| Bhat, 2016 | Puducherry, India;  January 2013-August 2013 | Controlled Intervention Study | Pediatric (<18 yrs.)  Comparator group: 183 patients Intervention group: 185 patients | NAAT | LDT | Conventional Microbiology | **LOS:** The hospital and NICU LOS significantly differed between the two groups (hospital LOS; 19 days in the control arm vs 15 days in the intervention arm, p-value <0.001. **NICU LOS:** 11 in the control arm vs 7 days in the intervention arm, p-value <0.001. **Mortality:** Mortality was significantly different between the two groups (n=33 in the intervention arm vs n=6 in the control arm, p-value <0.001) | ND | Government grant |
| Bhavsar, 2018 | New York City, New York, USA;  Comparator period: July 2014-June 2015 Intervention period: March 2016-February 2017 | Before-After (Pre-Post) Studies with No Control Group | Pediatric (<18 yrs.)  Comparator group: 210 patients Intervention group: 137 patients | Direct MALDI-TOF MS | FDA-approved | Conventional Microbiology | **TTT:** The mean time to effective therapy was not significantly different between the groups, p-value=0.282; However, the mean time to optimal therapy was significantly better with the intervention group, p-value = 0.0001. **LOS:** The mean infection-related length of stay was significantly better with the intervention group, days (SD) - Pre-MALDI 10.5 (7.6) & Post-MALDI 8.3 (7.0), p-value = 0.006. **Mortality:** The 30-day all-cause mortality significantly differed between groups, p-value = 0.496. | RUO MALDI Database; Direct MALDI (LDT) | Funding source not reported |
| Bhowmick, 2018 | New Brunswick, New Jersey, USA;  Comparator period: October 2013-January 2014 Intervention 1 period: February 2014-July 2014 Intervention 2 period: July 2014-October 2015  Intervention 3 period: November 2015-December 2017 | Before-After (Pre-Post) Studies With No Control Group | Adult  Comparator group: 55 patients Intervention group 1: 54 patient Intervention group 2: 123 patients  Intervention group 3: 126 patients | NAAT | FDA-approved | Conventional Microbiology | **TTT:** Time to directed therapy for MSSA and coagulase-negative Staphylococci decreased from 48.5 hrs. to 17.9 hrs. between the comparator period and the intervention 3 period, p-value =0.0015. | ND | No Funding |
| Box, 2015 | San Diego, CA, USA;  Comparator period: 2011 Intervention period: 2014 | Before-After (Pre-Post) Studies With No Control Group | Adult  Comparator group: 103 patients Intervention group: 64 patients | Microarray | FDA-approved | Conventional Microbiology | **TTT:**  The intervention group saw an improved mean time to targeted antibiotic therapy (61.1 vs 35.4 hrs., p-value <0.001). **LOS:** The median length of stay (9.1 vs 7.2 days, p-value = 0.04) was lower in the intervention group. **Mortality:** Mortality was similar between groups (9.1% vs 9.2%, p-value = 0.98). | Added call to Pharmacist in addition to microarray | Manufacturer/industry funding |
| Carreno, 2016 | Albany, New York, USA;  Comparator period: February 2013-August 2013 Intervention period: February 2014-August 2014 | Before-After (Pre-Post) Studies with No Control Group | Adult  Comparator group: 115 patients Intervention group: 104 patients | Direct MALDI-TOF MS and NAAT | FDA Approved and LDT | LDT and conventional microbiology | **TTT:** Time to activity therapy, median days (IQR) - comparator group, 0.30 days (0.06-0.87) & intervention group, 0.12 days (0.04-0.68), p-value = 0.015. **LOS:** There was not a significant difference between groups. Hospital length of stay, days (IQR) - comparator group, 9 days (4-15) & intervention group, 10 days (6-16), p-value = 0.249; ICU length of stay, days (IQR) - comparator group, 4 (2-7) & intervention group, 5 days (2-8), p-value = 0.363. **Mortality:** There was no significant difference in Inpatient mortality between groups, No. (%) - comparator group, 13 (11.4) & intervention group, 8 (7.6), p-value = 0.342. | ND | Hospital or hospital system |
| Carreno, 2019 | Albany, New York, USA;  Comparator period: January-December 2013 Intervention 1 period: January-December 2014 Intervention 2 period: July-December 2015 | Before-After (Pre-Post) Studies with No Control Group | Adult  Comparator group: 157 patients Intervention group 1: 176 patients Intervention group 2: 61 patients | Group 1: NAAT & direct MALDI-TOF MS Group 2: NAAT, direct MALDI-TOF MS and ASP notification | FDA Approved and LDT | Conventional Microbiology | **TTT:** Time to first active antibiotic, days; median (IQR) was 0.18 days (IQR: 0.05–0.73) for the comparator group, 0.09 days (IQR: 0.01–0.37) for intervention group 1 and 0.07 days (IQR:0.00–0.38) for intervention group 2 (p-value = 0.01). **LOS:** Hospital length of stay differed significantly among the three groups (9, 10, and 14 days), with intervention group 2 having the longest LOS (p-value = 0.01).  **Mortality:** No appreciable difference in risk of inpatient mortality among the 3 study groups (p-value = 0.17). | The prevalence of “unknown source or other” sources of infection was significantly higher in the intervention group. The “unknown source or other” sources of infection group was also associated with increased hospital length of stay (29 vs. 9 days, p = 0.003). | Not for profit |
| Cattoir, 2011 | Creteil, France, USA;  2006-2007 | Cohort or Cross-sectional Study | Adult & Pediatric (<18 yrs.)  Comparator group: 48 patients Intervention group:49 patients | NAAT | LDT | Conventional Microbiology | **TTT:** Time to initiation of appropriate therapy after final identification of staphylococci (hrs.) - comparator group, 27 hrs. (0-43) & intervention group, four hrs. (0-48), p-value = 0.012 |  | No Funding |
| Cosgrove, 2016 | Baltimore, Maryland;  2008-2009 | Controlled Intervention Study | Adult  220 blood cultures grew GPCs. Of these, 114 (52%) were randomized to the control arm and 106 (48%) to the PNA FISH arm. | PNA-FISH | FDA-modified, manufacturer's instructions were followed with the exception of the hybridization step in which a 30-minute, instead of a 90-minute incubation period was used. | Conventional Microbiology | **TTT:** Median times to effective therapy were 0 hrs. in PNA FISH and control groups (P-value = 0.25). Median times to optimal therapy were 12.1 hrs. (IQR 0.0–51.8) and 18.1 hrs. (IQR 0.0–50.6) in the PNA FISH and control groups, respectively (p-value = 0.92). **LOS:** The median length of hospital stay for the overall sample was 17 days in both groups (P= 0.65). **Mortality:** Both study groups had 19% all-cause in-hospital mortality (p-value = 0.94). |  | Manufacturer/industry |
| Delport, 2016 | London, Ontario;  Comparator period: 2012 Intervention 1 period: 2013 Intervention 2 period: 2014 | Before-After (Pre-Post) Studies with No Control Group | Adult & Pediatric (<18 yrs.)  Comparator group: 134 patients Intervention group 1: 143 patients  Intervention group 2: 117 patients | 3-hour short incubation MALDI-TOF MS | FDA-modified MALDI-TOF with a short incubation | Conventional Microbiology | **TTT:** Improvement in initiation of antibiotics of 3.33 hrs. between 2012 and 2014 (p-value <0.02). The time to antibiotic change was reduced by 6.94 hrs. (p-value <0.30) in 2013, but in the third arm in 2014, patients received more appropriate antibiotics 13.3 hrs. earlier (p-value <0.02). **LOS:** Overall, LOS was reduced by 4.54 days after short incubation of the MALDI-TOF identification protocol compared to conventional identification methods (p-value <0.031). | 3 study groups; only the rapid arm was a 3-hour shorter incubation before MALDI-TOF identification | Explicit statement of no funding received |
| Eby, 2018 | Charlottesville, Virginia, USA;  December 2012-July 2015. The exact period of the comparator and intervention groups was not indicated. | Before-After (Pre-Post) Studies with No Control Group | Adult  Comparator group: 106 patients Intervention group: 120 patients | Microarray | FDA Approved | PNA-FISH | **TTT:** The percent of patients on active antibiotic within 24 hrs. of Gram stain result was not statistically significant (98.0% preintervention vs 98.2% postintervention); percent to institutionally preferred staphylococcal therapy within 24 hrs. of Gram stain was increased from 21.2 to 86.4% (p-value <0.001).  **Mortality:** In-hospital mortality was higher in the comparator group than in the intervention group (p-value = 0.047. 30-day all-cause mortality was higher in the comparator group than in the intervention group (p-value =0.025). | *Staphylococcus aureus* bacteremia only | Funding source not reported |
| Farfour, 2019 | Suresnes, France;  May 2016-October 2016. The exact time period of the comparator and intervention groups were not indicated. | Before-After (Pre-Post) Studies with No Control Group | Adult  Comparator group: 42 patients  Intervention group: 41 patients | NAAT; Direct MALDI TOF MS; rapid susceptibility testing | CE-Marked | MALDI-TOF MS; conventional AST | **TTT:** There was no significant difference between groups. Effective therapy on day 1 - Comparator: 73.8%, Intervention: 82.9%, p-value = 0.43; appropriate therapy on day 1) - Comparator: 54.7%, Intervention: 70.7%, p-value = 0.17. **LOS:** There was no significant difference in the median LOS between the groups, comparator group: 10 days (6-16), intervention group: 7 days (5-12), p-value = 0.27. **Mortality:** There was no significant difference between the comparator group (9.5%) and intervention group (4.9%), p-value = 0.67. |  | Explicit statement of no funding received |
| Felsenstein, 2016 | Los Angeles, CA, USA;  Comparator period: October 2011-January 2013 Intervention period: February 2013-February 2014 | Before-After (Pre-Post) Studies with No Control Group | Pediatric (<21 yrs.)  Comparator group: 221 blood cultures from 194 patients Intervention group: 219 blood cultures from 189 patients | Microarray | FDA-approved | Conventional Microbiology | **TTT:** The TTT from Gram stain notification was shorter in the intervention group (34.1 vs 46.6 hrs.; p-value <0.006). **LOS:** No difference in hospital LOS between groups (16.1 days, comparator vs. 13.6 days intervention, p-value <0.35). **Mortality:** Crude mortality within 1 month of diagnosis of BSI had as lower mortality trend after with the intervention group, but was not significant (11 of 194 [5.7%] versus 5 of 189 [2.6%]; p-value = 0.28). |  | Explicit statement of no funding received |
| Forrest, 2006 | Baltimore, Maryland, USA;  The time frame was not defined. | Cohort or Cross-sectional Study | Adult Comparator group: 34 patients Intervention group: 53 patients | PNA-FISH | FDA-approved | Conventional Microbiology | **LOS:** Median length of stay (days) was found to be significant in the intervention group (6 days vs. 4 days, p-value <0.05). **Mortality:** There was no significant difference between the control and intervention groups (p-value = 0.64). |  | Funding source not reported. |
| Forrest, 2008 | Baltimore, Maryland, USA;  2005-2006 | Cohort or Cross-sectional Study | Adult and Pediatric  Comparator group: 129 patients Intervention group: 95 patients | PNA-FISH | FDA-approved | Conventional Microbiology | **TTT:**  In the *E. faecalis* group, the TTT was not statistically significant (0 vs 0.3 days); however, in the *E. faecium* group, 3.1 vs 1.3 days was (p-value <0.001). **LOS:** There was no statistically significant difference in either organism between the comparator and intervention groups. **Mortality:** 30-day mortality was higher in the comparator *E. faecium* group (29%) than the intervention group (12%),p-value =0 .039. In the *E. faecalis* group it was not statistically significant between the groups (8% vs 5%, p-value = 0.73). |  | University Hospital or hospital system |
| Frye, 2012 | Portland, Oregon, USA;  Comparator period: January 2009-January 2010 Intervention period: January 2010-January 2011 | Before-After (Pre-Post) Studies with No Control Group | Adult  Comparator group: 68 S. aureus and 66 Coagulase-negative staphylococci,  Intervention group: 58 *S. aureus* and 52 Coagulase-negative staphylococci | NAAT | FDA-approved | Conventional Microbiology | **TTT:** For MRSA, the time to optimal antibiotic therapy increased in the intervention group by 3.7 hrs. (p-value >0.1). For MSSA, there was no statistically significant change. **LOS:** There was no significant difference between groups. 12.1 days for the comparator group and 11.6 days for the intervention group, and it was not statistically significant (p-value >0.01).  **Mortality:** 17% mortality in the comparator group and 14% in the intervention group was not statistically significant. (p-value >0.01). | Batched PCR results | Explicit statement of no funding received |
| Gray, 2018 | Charlottesville, Virginia, USA; Comparator group: January 2012-April 2014 Intervention 1 period: April 2014-August 2014 Intervention 2 period: September 2014-December 2015 | Before-After (Pre-Post) Studies with No Control Group | Adult  Comparator group: 63 patients Intervention group 1: 13 patients Intervention group 2: 34 patients | Microarray with and without ASP notification | FDA-approved | PNA-FISH | **TTT:** Only analyzed time to institutional preferred therapy and showed from baseline and microarray plus communication (p-value = 0.02). Microarray and Microarray plus communication (p-value =0.04) times were significant for *E. faecium* only. **LOS:** Length of stay was significantly shorter for the microarray plus communication than baseline (p-value = 0.04). **Mortality:** No significant difference in mortality between cohorts. | Enterococcus only | Government |
| Heil, 2012 | Chapel Hill, North Carolina, USA;  Comparator group: June 2009-September 2010 Intervention group: September 2010-June 2011 | Before-After (Pre-Post) Studies with No Control Group | Adult  Comparator group: 61 patients Intervention group: 21 patients | PNA-FISH | FDA-approved | Conventional Microbiology | **TTT:** The intervention group was significantly better for the mean time to targeted therapy, days - Comparator: 2.3 days (95% CI 1.4 to 3.2) & Intervention: 0.6 days (95% CI -0.01 to 1.16), p-value = 0.0016. **LOS:** There was no significant difference between groups, median (IQR) length of stay, days - Comparator: 25 days (4 to 7) & Intervention: 12 days (9 to 30), p-value = 0.82. **Mortality:** There was no significant difference in mortality between groups, No. (%) - Comparator: 19 (31%) \| Intervention: 5 (24%), p-value >0.99. | The sample size was small. Single center Before & after the design was used. | Funding source not reported. |
| Herrera, 2019 | Albuquerque, New Mexico, USA;  Comparator period: January 2011-December 2011 Intervention period: January 2014-December 2014 | Before-After (Pre-Post) Studies with No Control Group | Pediatric (<18 yrs.)  Comparator group: 104 patients  Intervention group: 57 patients | Direct MALDI-TOF MS | FDA-approved | Conventional Microbiology | **TTT:** No significant difference was observed for TTT (36.5 h vs 40.2 h intervention). **LOS:** No significant difference was observed for LOS (1022 h vs 736.6 h post-intervention). **Mortality:** No difference in mortality was observed between the two groups (9.1% vs 8.2% post-intervention). |  | No external funding |
| Holtzman, 2011 | Boston, Massachusetts, USA;  Comparator period: May 2005-October 2006 Intervention period: December 2006-May 2008 | Before-After (Pre-Post) Studies with No Control Group | Randomly selected (age not reported)  Comparator group: 100 patients Intervention group: 99 patients | PNA-FISH | FDA-approved | Not reported | **LOS:** Length of stay was not significant between the comparator group (18.7 days) and the intervention group (20.9 days), p-value = 0.35. |  | Hospital or hospital system |
| Idelevich, 2015 | Münster, Germany;  May 2010-September 2012 | Controlled Intervention Study | Adult  Comparator group: 76 patients Intervention group: 74 patients | NAAT | CE-Marked | Conventional Microbiology | **TTT:** The median time to a targeted therapy was significantly shorter in the intervention group (21.4 hrs., range 16.2-46.3) than the comparator group (47.5 hrs., range 7.3-59.2 h), p-value =0.018. **LOS:**  there was no significant difference in the length of hospital or ICU stay.  **Mortality:** there was no significant difference in mortality between the comparator and intervention groups. | The SeptiFast was pulled from the market due to false-positive results (i.e., the assay was too sensitive). They found 32 instances where Staph. aureus was found by PCR but not by culture (so-called DNAemia). | Manufacturer/industry |
| Koncelik, 2016 | Winter Haven, Florida, USA; Comparator period: April 2013-August 2013 Intervention period: August 2013-January 2014 | Before-After (Pre-Post) Studies With No Control Group | Not Reported  Comparator group: 36 patients Intervention group: 26 patients | PNA-FISH | FDA Approved | Conventional Microbiology | **LOS:** There was no significant difference in the mean length of stay between the comparator and intervention groups (9.78 days, range 1-41 vs. 7.50 days, range 1-26), p-value = 0.2613. However, when the data was adjusted for patients who remained in-house for 10 days or less, a significant decrease was seen with the intervention group (4.89 days, range 1-10 vs. 3.44 days, range 1-9), p-value = 0.0484. | The sample size was small. Single center | Hospital or hospital system |
| Lockwood, 2016 | Houston, Texas, USA;  Comparator period: January 2011-December 2011  Intervention period: January 2014-December 2014 | Before-After (Pre-Post) Studies with No Control Group | Adult  Comparator group: 151 patients Intervention group: 242 patients | Direct MALDI-TOF MS + ASP notification | modified FDA | Conventional Microbiology | **TTT:** The time to therapy adjustment was markedly reduced from 75 (±59) hrs. to 30 (±30) hrs. (p-value < 0.001). Therapy adjustment was defined as de-escalation and/or escalation of antibiotic therapy, dosing and/or administration route modifications, and/or discontinuation of unnecessary Gram-positive coverage. **LOS:**  There was no significant difference between the groups regarding the length of stay. **Mortality:** Overall mortality did not differ significantly between the groups (9.4% vs 4.9%, p-value = 0.07). | Retrospective data Prescription prescribing practices was not evaluated between the two hospitals | Hospital or hospital system |
| MacVane, Hurst, 2016 | Charleston, South Carolina, USA Comparator period: January 2010-November 2013 Intervention period: December 2013-December 2014 | Before-After (Pre-Post) Studies with No Control Group | Adult  Comparator group: 45 patients Intervention group: 23 patients | NAAT + ASP notification | FDA Approved | Conventional Microbiology + ASP notification | **TTT:**  Time to effective therapy was significant in the intervention group (50.3 hrs. versus 20.8 hrs., p-value < 0.001. **LOS:** There was no significant difference between the groups. Comparator group: 23.0 days (13.5-45.0) vs. Intervention group: 23.0 days (7.0-34.0), p-value = 0.345. **Mortality:** There was no significant difference between the groups. | Refer to the Risk of Bias table. Only VRE was evaluated. | Hospital or hospital system (Internally Supported Study) |
| MacVane, Nolte, 2016 | Charleston, South Carolina, USA;  Comparator period: August-October, 2010 Intervention period 1: August-October, 2012 Intervention period 2: August-October, 2014 | Before-After (Pre-Post) Studies with No Control Group | Adult  Comparator group: 115 Intervention group 1: 104 Intervention group 2: 145 | Group 1: Conventional Microbiology + ASP notification Group2: NAAT + ASP notification | FDA Approved | Conventional Microbiology | **TTT:** Intervention group 2 had a shorter time to effective therapy (5 h; p-value < 0.001) than the comparator group (15 h) or intervention group 1 (13 h).  **LOS:** There was no significant difference between the groups. **Mortality:** There was no significant difference between the groups. |  | Manufacturer/industry |
| Magarifuchi, 2018 | Saga, Japan;  September 2011-February 2013 | Before-After (Pre-Post) Studies with No Control Group | Adult  Comparator group: 129 Intervention group: 119 | Direct MALDI-TOF MS + Disk Diffusion | FDA Approved | Conventional Microbiology | **Mortality:** 28-day mortality was not different between the two groups (14.8% vs 16.8% post-intervention). |  | Funding source not reported. |
| Neuberger, 2008 | Haifa, Israel; 2006 (9-month period) | Controlled Intervention Study | Adult  Comparator group: 42 patients Intervention group: 42 patients | NAAT | LDT | Conventional Microbiology | **TTT:** The use of NAAT was the only significant factor in decreasing the time to the initiation of appropriate antimicrobial therapy (hazards ratio, 3.03; confidence interval, 1.62 to 5.68; p-value = 0.01). **LOS:** There was no significant difference between the groups. **Mortality:** There was no significant difference between the groups. |  | No external funding was received. |
| Neuner, 2016 | Cleveland, Ohio, USA; Comparator period: February 2014-September 2014 Intervention period: September 2014-February 2015 | Before-After (Pre-Post) Studies With No Control Group | Adult  Comparator group: 300 patients Intervention group: 213 patients | Microarray + ASP notification | FDA Approved | Conventional Microbiology; PNA FISH | **TTT:** Time to optimal therapy in MSSA infections was significantly shorter in the intervention group than in the comparator group: 20 hrs. versus 52 hrs., respectively (p-value = 0.001). **LOS:** There was no significant difference in the LOS between groups. **Mortality:** There was no significant difference between the groups. |  | Explicit statement of no funding received |
| Nguyen, 2010 | Palo Alto, California, USA; Comparator period: December 2007-May 2008  Intervention period: December 2008-May 2009 | Before-After (Pre-Post) Studies with No Control Group | Adult  Comparator group: 65 Intervention group: 94 | NAAT | LDT | Conventional Microbiology | **TTT:** Time to optimal therapy in MSSA infections was significantly shorter in the intervention group than in the comparator group: 2 days versus 5 days, respectively (p-value <0.0001). **LOS:**  Length of stay was significant between the comparator group (8 days, range 1 to 47 days) and the intervention group (5 days, range 0 to 42 days), p-value = 0.03. |  | Explicit statement of no funding received |
| Niwa, 2019 | Gifu, Japan; Comparator period: June 2016-January 2017  Intervention period: June 2017-January 2018 | Before-After (Pre-Post) Studies with No Control Group | Adult  Comparator group: 180 patient Intervention group: 186 patients | Direct MALDI TOF MS + ASP notification | FDA Approved | Conventional Microbiology | **TTT:** Direct MALDI-TOF MS in conjunction with ASP notification significantly decreased the time to optimal therapy compared to the control group (53.3 hrs. vs 91.7 hrs.), p-value <0.001. **LOS:** There was no significant difference between the groups. **Mortality:** There was no significant difference between the groups. |  | Funding source not reported. |
| Pardo, 2016 | Gainesville, Florida, USA;  Comparator period: January 2012–June 2013 Intervention period: August 2013-January 2014 | Before-After (Pre-Post) Studies with No Control Group | Adult  Comparator group: 252 patients Intervention group: 84 patients | NAAT | FDA Approved | Conventional Microbiology | **TTT:**  Patients with VRE bacteremia received active therapy 16 hours earlier when compared to the control group (p-value = 0.047).  **LOS:** LOS was significantly shorter in the intervention group at 2.9 days [2.1 days –4.4 days] vs. the comparator group at 2.3 days [1.5 days –3.1 days], p-value =0.008.  **Mortality:** The intervention group was associated with a statistically significant reduction in mortality (p-value = 0.036). |  | Manufacturer/industry |
| Perez, 2013 | Houston, Texas, USA; Comparator period: August 2011-November 2011 Intervention period: February 2012-May 2012 | Before-After (Pre-Post) Studies with No Control Group | Adult  Comparator group: 112 Intervention group: 107 | Direct MALDI-TOF MS | Modified FDA Approved | Conventional Microbiology | **TTT:** Time to adjusted therapy (average time to the initiatiion of an active agent) was significant in the intervention group (comparator group 75 hrs. +/- 48 hrs. vs. intervention group at 29 hrs. +/- 17 hrs., p-value = 0.004). **LOS:** Hospital length of stay was significantly reduced in the intervention group (comparator 11.9 days +/- 9.3 days vs. Intervention 9.3 days +/- 7.6 days, p-value = 0.01). The hospital length of stay after BSI onset was significantly reduced in the intervention group (comparator 9.9 days +/- 7.1 days vs. Intervention 8.1 days +/- 6.4 days, p-value 0.01). **Mortality:** There was no significant difference in the all-cause 30-day mortality between the two groups, p-value = 0.19. |  | Funding source not reported. |
| Perez, 2014 | Houston, TX, USA;  Comparator period: January 2009-November 2011 Intervention period: February 2012-June 2013 | Before-After (Pre-Post) Studies with No Control Group | Adult  Comparator group: 157 Intervention group: 112 | Direct MALDI-TOF MS | Modified FDA Approved | Conventional Microbiology | **TTT:** The average time to optimal antimicrobial management in eligible patients was 80.9 h +/- 63 h during the comparator period compared with 23.2 +/- 19.9 h in the intervention group (128 and 96 patients, respectively, p-value < 0.001). **LOS:** The LOS was significantly shorter in the intervention group. The hospital length of stay was 23.3 days +/- 21.6 days for the comparator group and 15.3 days +/- 17.3 days for the intervention group, P-value = 0.0001. The mean hospital LOS for inpatient survivors in the comparator group was 23.3 days +/- 21.6 days and was significantly decreased to 15.3 days +/- 17.3 days in the intervention group, p-value = 0.0001. **Mortality:** 30-day and 60-day all-cause mortality were significantly lower in the intervention group (30-day: comparator group, 21% vs. intervention group, 8.9%, p-value = 0.01; and 60-day, comparator group, 30.6%) vs. intervention group, 12.5%,p-value =0.001). The inpatient mortality was also significantly lower in the intervention group (comparator group, 18.5% vs. intervention group, 8% ,p-value = 0.02). |  | Funding source not reported. |
| Rivard, 2017 | Cleveland, Ohio, USA;  Comparator period: January 2015-July 2015  Intervention period: July 2015-January 2016. | Before-After (Pre-Post) Studies with No Control Group | Adult  Comparator group: 456 Intervention group: 421 | Microarray + ASP notification | FDA Approved | Microarray without ASP notification | **TTT:** The median (IQR) time from Gram stain to antimicrobial switch was significantly decreased in the intervention group 28.6 hrs. (8.6 h–56.9 h) vs. the comparator group 44.1 hrs. (18.9 h–64.6 h), p-value = 0.0036. In patients on inactive antimicrobial therapy at the time of result, the median time from Gram stain to active therapy was lower in the intervention group, 8.8 hrs. (5.5 h–18.4h) versus 24.5 hrs. (4.9 h–44.3h) in the comparator group, p-value = 0.0344.  **LOS:** Hospital length of stay was decreased in the intervention group, 7 days (5 d–15d) versus 9 days (4.5 d–21 d), p-value = 0.001. **Mortality:** In-hospital mortality rate was similar between groups, 11.6% (intervention) versus 11.4% (comparator), p-value = 0.87. |  | Manufacturer/industry |
| Rodrigues, 2019 | Sao Paulo, Brazil:  October 2012-May 2016 | Controlled Intervention Study | Adult  Comparator group: 100 Intervention group: 100 | NAAT | CE-Marked | MALDI-TOF MS | **TTT:** The time to antimicrobial therapy adjustment was significantly decreased with the intervention group (8 h [7 h–14 ] ) vs. the comparator group (54 h [38 h–75h]), p-value < 0.001.  **LOS:** Post-infection length of stay showed no difference between the intervention and the comparator groups.  **Mortality:** There was no significant difference between the groups. |  | Not for profit |
| Romero-Gomez, 2017 | Madrid, Spain; Comparator period: July 2009-July 2010 Intervention period: November 2010-November 2011 | Before-After (Pre-Post) Studies with No Control Group | Adult  Comparator group: 133 patients Intervention: 94 patients | Direct MALDI-TOF MS + NAAT | FDA Approved | Conventional Microbiology | **TTT:** Time to targeted therapy was not evaluated. However, the intervention group (81.33%) showed a significant increase in the rate of antibiotic optimization in comparison to the comparator group (48.71%), p-value <0.0001 **LOS:** There was no significant difference between the groups. Comparator group: 28.27 days (+32.16) vs. Intervention group: 28.62 days (+28.75), p-value = 0.26. | Only *S. aureus* evaluated | Explicit statement of no funding received |
| Roshdy, 2015 | Chapel Hill, North Carolina, USA;  The comparator and intervention occurred over 17 months; the exact months and years were not indicated. | Before-After (Pre-Post) Studies with No Control Group | Adult  Comparator group: 65 patients Intervention: 74 patients | NAAT | FDA Approved | Conventional Microbiology | **TTT:** Mean (SD) time to appropriate antibiotic was significantly better with the intervention group (comparator was 10.2 hrs. (19.8 hrs.) in comparison to the intervention group, which was 4.5 hrs. (14.4 hrs.), p-value =0.07; the mean (SD) time to acceptable antibiotic therapy was significantly better in the intervention group (comparator group was 13.2 hrs. (46.0 hrs.) vs. the comparator group was 1.9 hrs. (7.2 hrs.), p-value = 0.04).  **LOS:** There was no significant change in the LOS when the intervention group was compared to the comparator group.  **Mortality:** There was no difference in mortality (p-value =0.82) between the two groups. |  | Industry |
| Seo, 2018 | New York, NY, USA; Comparator period: January 2011-December 2011 Intervention period: January 2013-December 2013 | Before-After (Pre-Post) Studies with No Control Group | Adult  Comparator: 196 patients Intervention: 103 patients | PNA FISH | FDA Approved | Conventional Microbiology | **LOS:** There was no difference in LOS (p-value = 0.19) between the two groups. **Mortality:** There was no difference in mortality (p-value = 0.46) between the two groups. | Only CoNS evaluated | Government grant |
| Sothoron2015 | Jacksonville, Florida, USA;  Comparator period: September 2013-February 2014 Intervention period: September 2014-February 2015 | Before-After (Pre-Post) Studies with No Control Group | Adult  Comparator: 58 patients Intervention: 58 patients | NAAT + ASP notification | FDA Approved | Conventional Microbiology | **TTT:** The median time to optimal therapy was shorter in the intervention group, 49.3 h [95% confidence interval {CI}, 41.7, 65.0] in comparison to the comparator arm group,38.5 h [95% CI, 28.0, 45.6]; p-value = 0.0199.  **LOS:** There was no significant difference between the groups (mean hospital length of stay, p-value; =0.2055; mean infection-related length of stay, p-value = 0.9143).  **Mortality: T**here was no difference in mortality (p-value 0.9837) between the two groups. |  | No external funding. |
| Suzuki, 2015 | Tsukuba, Japan;  Comparator period: October2012-September 2013 Intervention period: July 2014-December 2014 | Before-After (Pre-Post) Studies with No Control Group | Adult  Comparator: 147 patients Intervention: 88 patients | Microarray | FDA Approved | Conventional Microbiology | **TTT:**  Time to appropriate antibiotic therapy was significantly shorter with the intervention group, p-value = 0.001. **Mortality:** 14-day mortality was not different between the two groups (5.4% comparator vs. 1.1% intervention). 30-day mortality was lower in the intervention group (12.9% comparator vs. 3.4% intervention, p-value = 0.019). |  | Industry |
| Tafelski, 2015 | Berlin, Germany;  August 2010-March, 2012 | double-blind, parallel-group randomized control trial | Adult  Comparator: 50 patients Intervention: 50 patients | NAAT | CE-Marked | Conventional Microbiology | **TTT:** Adaptation to appropriate antibiotic therapy was earlier in the intervention group (38.3 hrs, comparator vs. 18.8 hrs, intervention). **LOS:** LOS was not different between the two groups (37 days v.s 53 days, p-value >0.05). **Mortality:** ICU mortality was not different between the two groups (22% vs 17% intervention, p-value >0.05). |  | Industry |
| Tseng, 2018 | Scottsdale, Arizona, USA; Comparator period: June 2015-December 2015 Intervention period: June 2016-December 2016) | Before-After (Pre-Post) Studies with No Control Group | Adult  Comparator group: 103 patients Intervention group: 100 patients | NAAT | FDA Approved | Conventional Microbiology | **TTT:** The intervention arm did not lead to significant changes in antimicrobial therapy during the assessed period. There were no significant differences between appropriate escalation and antimicrobial therapy de-escalation times. **LOS:** There were no significant differences in the length of stay between the two groups. **Mortality:** There were no significant differences in hospital mortality between the comparator and the intervention groups. |  | Explicit statement of no external funding received |
| Turner, 2017 | Portland, Oregon, USA; Comparator period: April 2012-April 2014 Intervention period: May 2014-June 2015 | Before-After (Pre-Post) Studies with No Control Group | Adult  Comparator group: 343 patients Intervention group: 130 patients | NAAT | FDA Approved | Conventional Microbiology | **TTT:** Time to initiate optimal therapy was shorter in the PCR group for those infected with MSSA (median 40.0 h vs. 28.3 h, P = 0.001). After controlling for confounding factors, including infectious diseases consultation, the PCR group had a shorter time to initiate optimal therapy by 9.7 h (95% CI 4.3–15.0 h). **LOS:** There were no significant difference in the median length of stay between the two groups, p-value = 0.22.  **Mortality:** There were no significant differences in the all-cause, in-hospital 30-day mortality when both groups were evaluated, p-value = 0.63. |  | Explicit statement of no external funding received |
| Vlek, 2012 | Utrecht, Netherlands; Comparator period: December 2009-March 2010 Intervention period: February 2010-April 2010 | Before-After (Pre-Post) Studies with No Control Group | Adult  Comparator group: 164 patients Intervention group: 89 patients | Direct MALDI-TOF MS | FDA Modified | Conventional Microbiology | **TTT:** Median times until the first switch in antibiotic therapy were 17.5 hrs. (IQR 9.8–38.8) in the intervention group and 24.0 hrs. (IQR 9.5–47.0) in the comparator group, p-value = 0.30. Twenty-four hrs. after blood culture positivity, appropriate treatment was observed in 64.0% of the comparator group and 75.3% of the intervention group, p-value = 0.01. |  | Explicit statement of no external funding received. |
| Walker, 2016 | Los Angeles, California, USA;  Comparator period: May 2013-November 2013  Intervention period: December 2014-May 2015 | Before-After (Pre-Post) Studies with No Control Group | Adult  Comparator group: 98 patients Intervention group: 97 patients | Microarray | FDA Approved | Conventional Microbiology | **LOS:** The mean LOS was not different between the two groups (15.2 days, comparator vs 18.0 days, intervention).  **Mortality:** The 30-day mortality significantly decreased in the intervention group (19.2%, comparator vs. 8% post-intervention; p-value = 0.04). |  | Funding source not reported. |

Abbreviations: ASP, antimicrobial stewardship program; LDT, laboratory developed test; LOS, length of stay; MALDI-TOF MS, matrix assisted laser desorption ionization - time of flight mass spectrometry; PBP-2, penicillin binding protein-2; NAAT, nucleic acid amplification; ND, not determined; PNA FISH, peptide nucleic acid fluorescence in situ hybridization; TTT, time to targeted therapy

**Table 2S. Subgroup Analysis for Risk of Bias^a^ by Outcome**

| **Subgroup** | **N Studies** | **MD^b^** | **MD**  **LCI** | **MD**  **UCI** | **Subgroup Comparison p-value (*Significant subgroup comparison)** |
| --- | --- | --- | --- | --- | --- |
| **TTT** |  |  |  |  | 0.9167 |
| Low Risk | 28 | -17.27 | -25.73 | -8.81 | * |
| Moderate Risk | 2 | -23.85 | -66.36 | 18.65 |  |
| High Risk | 8 | -19.52 | -19.52 | -9.02 | * |
| **LOS** |  |  |  |  | 0.3927 |
| Low Risk | 31 | -2.16 | -3.60 | -0.71 | * |
| Moderate Risk | 2 | 6.66 | -6.21 | 19.52 |  |
| High Risk | 12 | -2.38 | -4.05 | -0.72 | * |
|  | **N Studies** | **RR** | **RR**  **LCI** | **RR**  **UCI** | **Subgroup Comparison p-value (*Significant subgroup comparison)** |
| **Mortality** |  |  |  |  | 0.5291 |
| Low Risk | 32 | 0.75 | 0.63 | 0.88 | * |
| Moderate Risk | 2 | 0.89 | 0.60 | 1.33 |  |
| High Risk | 11 | 0.86 | 0.68 | 1.09 |  |

^a^ Bias: may arise from publication bias, a phenomenon where studies with uninteresting or unfavorable results are less likely to be published than those with more favorable results, or other forms of bias, including a population that is non-representative of the overall population, confounding factors such as differential study quality, and other sources of bias.

^b^MD: Mean Difference

^c^RR: Risk ratio

*Significant effect at the subgroup level

**REFERENCES**

1. **Alvarez J, Mar J, Varela-Ledo E, Garea M, Matinez-Lamas L, Rodriguez J, Regueiro B.** 2012. Cost analysis of real-time polymerase chain reaction microbiological diagnosis in patients with septic shock. *Anaesth Intensive Care* **40:**958-963.
2. **Avdic E, Wang R, Li DX, Tamma PD, Shulder SE, Carroll KC, Cosgrove SE.** 2017. Sustained impact of a rapid microarray-based assay with antimicrobial stewardship interventions on optimizing therapy in patients with Gram-positive bacteraemia. *J Antimicrob Chemother* **72:**3191-3198.
3. **Banerjee R, Teng CB, Cunningham SA, Ihde SM, Steckelberg JM, Moriarty JP, Shah ND, Mandrekar JN, Patel R.** 2015. Randomized Trial of Rapid Multiplex Polymerase Chain Reaction-Based Blood Culture Identification and Susceptibility Testing. *Clin Infect Dis* **61:**1071-1080.
4. **Bauer KA, West JE, Balada-Llasat JM, Pancholi P, Stevenson KB, Goff DA.** 2010. An antimicrobial stewardship program's impact with rapid polymerase chain reaction methicillin-resistant Staphylococcus aureus/S. aureus blood culture test in patients with S. aureus bacteremia. *Clin Infect Dis* **51:**1074-1080.
5. **Beuving J, Wolffs PF, Hansen WL, Stobberingh EE, Bruggeman CA, Kessels A, Verbon A.** 2015. Impact of same-day antibiotic susceptibility testing on time to appropriate antibiotic treatment of patients with bacteraemia: a randomised controlled trial. *Eur J Clin Microbiol Infect Dis* **34:**831-838.
6. **Bhat BV, Prasad P, Ravi Kumar VB, Harish BN, Krishnakumari K, Rekha A, Manjunath G, Adhisivam B, Shruthi B.** 2016. Syndrome Evaluation System (SES) *versus* Blood Culture (BACTEC) in the Diagnosis and Management of Neonatal Sepsis - A Randomized Controlled Trial. *Indian J Pediatr* **83:**370-379.
7. **Bhavsar SM, Dingle TC, Hamula CL.** 2018. The impact of blood culture identification by MALDI-TOF MS on the antimicrobial management of pediatric patients. *Diagn Microbiol Infect Dis* **92:**220-225.
8. **Bhowmick T, Kirn TJ, Hetherington F, Takavarasha S, Sandhu SS, Gandhi S, Narayanan N, Weinstein MP.** 2018. Collaboration between an antimicrobial stewardship team and the microbiology laboratory can shorten time to directed antibiotic therapy for methicillin-susceptible staphylococcal bacteremia and to discontinuation of antibiotics for coagulase-negative staphylococcal contaminants. *Diagn Microbiol Infect Dis* **92:**214-219.
9. **Box MJ, Sullivan EL, Ortwine KN, Parmenter MA, Quigley MM, Aguilar-Higgins LM, MacIntosh CL, Goerke KF, Lim RA.** 2015. Outcomes of rapid identification for gram-positive bacteremia in combination with antibiotic stewardship at a community-based hospital system. *Pharmacotherapy* **35:**269-276.
10. **Carreno JJ, Eaton R, Itro L, Babowicz F, Falvo J, Tobin E, Mitchell C, George M.** 2019. Time to clinical response in sepsis associated with an algorithm for blood-culture pathogen identification using matrix-assisted laser desorption ionization time-of-flight mass spectroscopy. *Am J Health Syst Pharm* **76:**460-469.
11. **Carreno JJ, Lomaestro BM, Jacobs AL, Meyer RE, Evans A, Montero CI.** 2016. Assessment of Time to Clinical Response in Patients with Sepsis Treated Before and After Implementation of a Matrix-Assisted Laser Desorption Ionization Time-of-Flight Blood Culture Identification Algorithm. *Infect Control Hosp Epidemiol* **37:**916-923.
12. **Cattoir V, Merabet L, Djibo N, Rioux C, Legrand P, Girou E, Lesprit P.** 2011. Clinical impact of a real-time PCR assay for rapid identification of Staphylococcus aureus and determination of methicillin resistance from positive blood cultures. *Clin Microbiol Infect* **17:**425-431.
13. **Cosgrove SE, Li DX, Tamma PD, Avdic E, Hadhazy E, Wakefield T, Gherna M, Carroll KC.** 2016. Use of PNA FISH for blood cultures growing Gram-positive cocci in chains without a concomitant antibiotic stewardship intervention does not improve time to appropriate antibiotic therapy. *Diagn Microbiol Infect Dis* **86:**86-92.
14. **Delport JA, Strikwerda A, Armstrong A, Schaus D, John M.** 2016. Quality of Care Is Improved by Rapid Short Incubation MALDI-ToF Identification from Blood Cultures as Measured by Reduced Length of Stay and Patient Outcomes as Part of a Multi-Disciplinary Approach to Bacteremia in Pediatric Patients. *PLoS One* **11:**e0160618.
15. **Eby JC, Richey MM, Platts-Mills JA, Mathers AJ, Novicoff WM, Cox HL.** 2018. A Healthcare Improvement Intervention Combining Nucleic Acid Microarray Testing With Direct Physician Response for Management of Staphylococcus aureus Bacteremia. *Clin Infect Dis* **66:**64-71.
16. **Farfour E, Si Larbi AG, Cardot E, Limousin L, Mathonnet D, Cahen P, Vasse M, Lesprit P.** 2019. Impact of rapid diagnostic tests on the management of patients presenting with Enterobacteriaceae bacteremia. *Med Mal Infect* **49:**202-207.
17. **Felsenstein S, Bender JM, Sposto R, Gentry M, Takemoto C, Bard JD.** 2016. Impact of a Rapid Blood Culture Assay for Gram-Positive Identification and Detection of Resistance Markers in a Pediatric Hospital. *Arch Pathol Lab Med* **140:**267-275.
18. **Forrest GN, Mehta S, Weekes E, Lincalis DP, Johnson JK, Venezia RA.** 2006. Impact of rapid *in situ* hybridization testing on coagulase-negative staphylococci positive blood cultures. *J Antimicrob Chemother* **58:**154-158.
19. **Forrest GN, Roghmann MC, Toombs LS, Johnson JK, Weekes E, Lincalis DP, Venezia RA.** 2008. Peptide nucleic acid fluorescent in situ hybridization for hospital-acquired enterococcal bacteremia: delivering earlier effective antimicrobial therapy. *Antimicrob Agents Chemother* **52:**3558-3563.
20. **Frye AM, Baker CA, Rustvold DL, Heath KA, Hunt J, Leggett JE, Oethinger M.** 2012. Clinical impact of a real-time PCR assay for rapid identification of staphylococcal bacteremia. *J Clin Microbiol* **50:**127-133.
21. **Gray ME, Cox HL, Donohue LE, Poulter MD, Eby JC, Mathers AJ.** 2018. The effect of rapid diagnostic testing with Infectious Diseases fellow consultative intervention on the management of enterococcal bloodstream infection. *Diagn Microbiol Infect Dis* **92:**319-324.
22. **Heil EL, Daniels LM, Long DM, Rodino KG, Weber DJ, Miller MB.** 2012. Impact of a rapid peptide nucleic acid fluorescence in situ hybridization assay on treatment of Candida infections. *Am J Health Syst Pharm* **69:**1910-1914.
23. **Herrera L, Culbreath K, Dehority W.** 2019. Impact of Matrix-Assisted Laser Desorption/Ionization Time-of-Flight for the Identification of Gram-Positive Bloodstream Pathogens without Antimicrobial Stewardship Intervention in Hospitalized Children. *J Pediatr Infect Dis* **14:**144-148.
24. **Holtzman C, Whitney D, Barlam T, Miller NS.** 2011. Assessment of impact of peptide nucleic acid fluorescence in situ hybridization for rapid identification of coagulase-negative staphylococci in the absence of antimicrobial stewardship intervention. *J Clin Microbiol* **49:**1581-1582.
25. **Idelevich EA, Silling G, Niederbracht Y, Penner H, Sauerland MC, Tafelski S, Nachtigall I, Berdel WE, Peters G, Becker K, Molecular Diagnostics of Sepsis Study G.** 2015. Impact of multiplex PCR on antimicrobial treatment in febrile neutropenia: a randomized controlled study. *Med Microbiol Immunol* **204:**585-592.
26. **Koncelik DL, Hernandez J.** 2016. The Impact of Implementation of Rapid QuickFISH Testing for Detection of Coagulase-Negative Staphylococci at a Community-Based Hospital. *Am J Clin Pathol* **145:**69-74.
27. **Lockwood AM, Perez KK, Musick WL, Ikwuagwu JO, Attia E, Fasoranti OO, Cernoch PL, Olsen RJ, Musser JM.** 2016. Integrating Rapid Diagnostics and Antimicrobial Stewardship in Two Community Hospitals Improved Process Measures and Antibiotic Adjustment Time. *Infect Control Hosp Epidemiol* **37:**425-432.
28. **MacVane SH, Hurst JM, Boger MS, Gnann JW, Jr.** 2016. Impact of a rapid multiplex polymerase chain reaction blood culture identification technology on outcomes in patients with vancomycin-resistant Enterococcal bacteremia. *Infect Dis (Lond)* **48:**732-737.
29. **MacVane SH, Nolte FS.** 2016. Benefits of Adding a Rapid PCR-Based Blood Culture Identification Panel to an Established Antimicrobial Stewardship Program. *J Clin Microbiol* **54:**2455-2463.
30. **Magarifuchi H, Hamada Y, Oho M, Kusaba K, Urakami T, Aoki Y.** 2018. Clinical utility of direct application of matrix-assisted laser desorption ionization time-of-flight mass spectrometry and rapid disk diffusion test in presumptive antimicrobial therapy for bacteremia. *J Infect Chemother* **24:**881-886.
31. **Neuberger A, Oren I, Sprecher H.** 2008. Clinical impact of a PCR assay for rapid identification of Klebsiella pneumoniae in blood cultures. *J Clin Microbiol* **46:**377-379.
32. **Neuner EA, Pallotta AM, Lam SW, Stowe D, Gordon SM, Procop GW, Richter SS.** 2016. Experience With Rapid Microarray-Based Diagnostic Technology and Antimicrobial Stewardship for Patients With Gram-Positive Bacteremia. *Infect Control Hosp Epidemiol* **37:**1361-1366.
33. **Nguyen DT, Yeh E, Perry S, Luo RF, Pinsky BA, Lee BP, Sisodiya D, Baron EJ, Banaei N.** 2010. Real-time PCR testing for mecA reduces vancomycin usage and length of hospitalization for patients infected with methicillin-sensitive staphylococci. *J Clin Microbiol* **48:**785-790.
34. **Niwa T, Yonetamari J, Hayama N, Fujibayashi A, Ito-Takeichi S, Suzuki K, Ohta H, Niwa A, Tsuchiya M, Yamamoto M, Hatakeyama D, Hayashi H, Obara M, Sugiyama T, Baba H, Suzuki A, Murakami N.** 2019. Clinical impact of matrix-assisted laser desorption ionization-time of flight mass spectrometry combined with antimicrobial stewardship interventions in patients with bloodstream infections in a Japanese tertiary hospital. *Int J Clin Pract* **73:**e13332.
35. **Pardo J, Klinker KP, Borgert SJ, Butler BM, Giglio PG, Rand KH.** 2016. Clinical and economic impact of antimicrobial stewardship interventions with the FilmArray blood culture identification panel. *Diagn Microbiol Infect Dis* **84:**159-164.
36. **Perez KK, Olsen RJ, Musick WL, Cernoch PL, Davis JR, Land GA, Peterson LE, Musser JM.** 2013. Integrating rapid pathogen identification and antimicrobial stewardship significantly decreases hospital costs. *Arch Pathol Lab Med* **137:**1247-1254.
37. **Perez KK, Olsen RJ, Musick WL, Cernoch PL, Davis JR, Peterson LE, Musser JM.** 2014. Integrating rapid diagnostics and antimicrobial stewardship improves outcomes in patients with antibiotic-resistant Gram-negative bacteremia. *J Infect* **69:**216-225.
38. **Rivard KR, Athans V, Lam SW, Gordon SM, Procop GW, Richter SS, Neuner E.** 2017. Impact of antimicrobial stewardship and rapid microarray testing on patients with Gram-negative bacteremia. *Eur J Clin Microbiol Infect Dis* **36:**1879-1887.
39. **Rodrigues C, Siciliano RF, Filho HC, Charbel CE, de Carvalho Sarahyba da Silva L, Baiardo Redaelli M, de Paula Rosa Passetti AP, Franco MRG, Rossi F, Zeigler R, De Backer D, Franco RA, de Almeida JP, Rizk SI, Fukushima JT, Landoni G, Uip DE, Hajjar LA, Strabelli TMV.** 2019. The effect of a rapid molecular blood test on the use of antibiotics for nosocomial sepsis: a randomized clinical trial. *J Intensive Care* **7:**37.
40. **Romero-Gomez MP, Cendejas-Bueno E, Garcia Rodriguez J, Mingorance J.** 2017. Impact of rapid diagnosis of Staphylococcus aureus bacteremia from positive blood cultures on patient management. *Eur J Clin Microbiol Infect Dis* **36:**2469-2473.
41. **Roshdy DG, Tran A, LeCroy N, Zeng D, Ou FS, Daniels LM, Weber DJ, Alby K, Miller MB.** 2015. Impact of a rapid microarray-based assay for identification of positive blood cultures for treatment optimization for patients with streptococcal and enterococcal bacteremia. *J Clin Microbiol* **53:**1411-1414.
42. **Seo SK, Gedrimaite Z, Paskovaty A, Seier K, Morjaria S, Cohen N, Riedel E, Tang YW, Babady NE.** 2018. Impact of QuickFISH in addition to antimicrobial stewardship on vancomycin use and resource utilization in cancer patients with coagulase-negative staphylococcal blood cultures. *Clin Microbiol Infect* **24:**1339 e1337-1339 e1312.
43. **Sothoron C, Ferreira J, Guzman N, Aldridge P, McCarter YS, Jankowski CA.** 2015. A Stewardship Approach To Optimize Antimicrobial Therapy through Use of a Rapid Microarray Assay on Blood Cultures Positive for Gram-Negative Bacteria. *J Clin Microbiol* **53:**3627-3629.
44. **Tafelski S, Nachtigall I, Adam T, Bereswill S, Faust J, Tamarkin A, Trefzer T, Deja M, Idelevich EA, Wernecke KD, Becker K, Spies C, Molecular Diagnostics of Sepsis Study G.** 2015. Randomized controlled clinical trial evaluating multiplex polymerase chain reaction for pathogen identification and therapy adaptation in critical care patients with pulmonary or abdominal sepsis. *J Int Med Res* **43:**364-377.
45. **Suzuki H, Hitomi S, Yaguchi Y, Tamai K, Ueda A, Kamata K, Tokuda Y, Koganemaru H, Kurihara Y, Ishikawa H, Yanagisawa H, Yanagihara K.** 2015. Prospective intervention study with a microarray-based, multiplexed, automated molecular diagnosis instrument (Verigene system) for the rapid diagnosis of bloodstream infections, and its impact on the clinical outcomes. *J Infect Chemother* **21:**849-856.
46. **Tseng AS, Kasule SN, Rice F, Mi L, Chan L, Seville MT, Grys TE.** 2018. Is It Actionable? An Evaluation of the Rapid PCR-Based Blood Culture Identification Panel on the Management of Gram-Positive and Gram-Negative Blood Stream Infections. *Open Forum Infect Dis* **5:**ofy308.
47. **Turner RB, Lalikian K, Fry M, Schwartz J, Chan D, Won R.** 2017. Impact of rapid identification of Staphylococcus aureus bloodstream infection without antimicrobial stewardship intervention on antibiotic optimization and clinical outcomes. *Diagn Microbiol Infect Dis* **89:**125-130.
48. **Vlek AL, Bonten MJ, Boel CH.** 2012. Direct matrix-assisted laser desorption ionization time-of-flight mass spectrometry improves appropriateness of antibiotic treatment of bacteremia. *PLoS One* **7:**e32589.
49. **Walker T, Dumadag S, Lee CJ, Lee SH, Bender JM, Cupo Abbott J, She RC.** 2016. Clinical Impact of Laboratory Implementation of Verigene BC-GN Microarray-Based Assay for Detection of Gram-Negative Bacteria in Positive Blood Cultures. *J Clin Microbiol* **54:**1789-1796.
