# Supplementary material for: The American Society for Microbiology’s evidence-based laboratory medicine practice guidelines for the diagnosis of bloodstream infections using rapid tests: a systematic review and meta-analysis
Source: Clin Microbiol Rev. 2025 Jun 16;38(3):e00137-24. doi: 10.1128/cmr.00137-24 (PMC12424361; doi:10.1128/cmr.00137-24)
Supplement: Supplemental Table and Figure Legends [file cmr.00137-24-s0009.docx]

**SUPPLEMENTARY TABLES**

Table S1. Narrative Summary and Characteristics of Included Studies

Table 2S. Subgroup Analysis for Risk of Bias by Outcome

**SUPPLEMENTARY FIGURE LEGENDS**

Figure 1S. Algorithm to Determine the Formula to Estimate Means and Standard Deviations

Figure 2S. Risk of Bias by Study-Before-After Design

Figure 3S. Risk of Bias by Study - Cohort Designs

Figure 4S. Risk of Bias by Study - Controlled Study Designs

Figure 5S. Study Bias for Time-to-Targeted Treatment Outcomes. In Figure 5S, a contour-enhanced **sample-size-based funnel plot** is depicted to investigate publication bias and other biases in the meta-analysis of TTT. The plot depicts the study-specific total sample sizes **graphed against the corresponding effect size estimates, enhanced by grayscale contours representing** statistical significance**.** The white funnel is centered at 0, representing the value under the null hypothesis (no effect). In contrast, the funnel represented by the dotted line is centered on the model’s impact estimate, which was created by an analysis of studies of TTT. The shaded regions indicate different levels of statistical significance of the studies represented.

Figure 6S. Study Bias for Length of Stay Outcomes. In Figure 6S, the contour-enhanced **sample-size-based funnel plot** is depicted to investigate bias in the meta-analysis of LOS. Refer to Figure 5S for a description of the funnel plot.

Figure 7S. Study Bias for Mortality Outcomes. In Figure 7S, the contour-enhanced **sample-size-based funnel plot** is depicted to investigate bias in the meta-analysis of mortality. Refer to Figure 5S for a description of the funnel plot.

**REFERENCES**

**Fig. 1S**

1. **Cai S, Zhou J, Pan J.** 2021. Estimating the sample mean and standard deviation from order statistics and sample size in meta-analysis. *Stat Methods Med Res* **30:**2701-2719.
2. **Luo D, Wan X, Liu J, Tong T.** 2018. Optimally estimating the sample mean from the sample size, median, mid-range, and/or mid-quartile range. *Stat Methods Med Res* **27:**1785-1805.
3. **Wan X, Wang W, Liu J, Tong T.** 2014. Estimating the sample mean and standard deviation from the sample size, median, range and/or interquartile range. *BMC Med Res Methodol* **14:**135.

**Fig. 2S**

1. **Alvarez J, Mar J, Varela-Ledo E, Garea M, Matinez-Lamas L, Rodriguez J, Regueiro B.** 2012. Cost analysis of real-time polymerase chain reaction microbiological diagnosis in patients with septic shock. *Anaesth Intensive Care* **40:**958-963.
2. **Avdic E, Wang R, Li DX, Tamma PD, Shulder SE, Carroll KC, Cosgrove SE.** 2017. Sustained impact of a rapid microarray-based assay with antimicrobial stewardship interventions on optimizing therapy in patients with Gram-positive bacteraemia. *J Antimicrob Chemother* **72:**3191-3198.
3. **Bauer KA, West JE, Balada-Llasat JM, Pancholi P, Stevenson KB, Goff DA.** 2010. An antimicrobial stewardship program's impact with rapid polymerase chain reaction methicillin-resistant Staphylococcus aureus/S. aureus blood culture test in patients with S. aureus bacteremia. *Clin Infect Dis* **51:**1074-1080.
4. **Bhavsar SM, Dingle TC, Hamula CL.** 2018. The impact of blood culture identification by MALDI-TOF MS on the antimicrobial management of pediatric patients. *Diagn Microbiol Infect Dis* **92:**220-225.
5. **Bhowmick T, Kirn TJ, Hetherington F, Takavarasha S, Sandhu SS, Gandhi S, Narayanan N, Weinstein MP.** 2018. Collaboration between an antimicrobial stewardship team and the microbiology laboratory can shorten time to directed antibiotic therapy for methicillin-susceptible staphylococcal bacteremia and to discontinuation of antibiotics for coagulase-negative staphylococcal contaminants. *Diagn Microbiol Infect Dis* **92:**214-219.
6. **Box MJ, Sullivan EL, Ortwine KN, Parmenter MA, Quigley MM, Aguilar-Higgins LM, MacIntosh CL, Goerke KF, Lim RA.** 2015. Outcomes of rapid identification for gram-positive bacteremia in combination with antibiotic stewardship at a community-based hospital system. *Pharmacotherapy* **35:**269-276.
7. **Carreno JJ, Lomaestro BM, Jacobs AL, Meyer RE, Evans A, Montero CI.** 2016. Assessment of Time to Clinical Response in Patients with Sepsis Treated Before and After Implementation of a Matrix-Assisted Laser Desorption Ionization Time-of-Flight Blood Culture Identification Algorithm. *Infect Control Hosp Epidemiol* **37:**916-923.
8. **Carreno JJ, Eaton R, Itro L, Babowicz F, Falvo J, Tobin E, Mitchell C, George M.** 2019. Time to clinical response in sepsis associated with an algorithm for blood-culture pathogen identification using matrix-assisted laser desorption ionization time-of-flight mass spectroscopy. *Am J Health Syst Pharm* **76:**460-469.
9. **Delport JA, Strikwerda A, Armstrong A, Schaus D, John M.** 2016. Quality of Care Is Improved by Rapid Short Incubation MALDI-ToF Identification from Blood Cultures as Measured by Reduced Length of Stay and Patient Outcomes as Part of a Multi-Disciplinary Approach to Bacteremia in Pediatric Patients. *PLoS One* **11:**e0160618.
10. **Eby JC, Richey MM, Platts-Mills JA, Mathers AJ, Novicoff WM, Cox HL.** 2018. A Healthcare Improvement Intervention Combining Nucleic Acid Microarray Testing With Direct Physician Response for Management of Staphylococcus aureus Bacteremia. *Clin Infect Dis* **66:**64-71.
11. **Farfour E, Si Larbi AG, Cardot E, Limousin L, Mathonnet D, Cahen P, Vasse M, Lesprit P.** 2019. Impact of rapid diagnostic tests on the management of patients presenting with Enterobacteriaceae bacteremia. *Med Mal Infect* **49:**202-207.
12. **Felsenstein S, Bender JM, Sposto R, Gentry M, Takemoto C, Bard JD.** 2016. Impact of a Rapid Blood Culture Assay for Gram-Positive Identification and Detection of Resistance Markers in a Pediatric Hospital. *Arch Pathol Lab Med* **140:**267-275.
13. **Forrest GN, Roghmann MC, Toombs LS, Johnson JK, Weekes E, Lincalis DP, Venezia RA.** 2008. Peptide nucleic acid fluorescent in situ hybridization for hospital-acquired enterococcal bacteremia: delivering earlier effective antimicrobial therapy. *Antimicrob Agents Chemother* **52:**3558-3563.
14. **Frye AM, Baker CA, Rustvold DL, Heath KA, Hunt J, Leggett JE, Oethinger M.** 2012. Clinical impact of a real-time PCR assay for rapid identification of staphylococcal bacteremia. *J Clin Microbiol* **50:**127-133.
15. **Gray ME, Cox HL, Donohue LE, Poulter MD, Eby JC, Mathers AJ.** 2018. The effect of rapid diagnostic testing with Infectious Diseases fellow consultative intervention on the management of enterococcal bloodstream infection. *Diagn Microbiol Infect Dis* **92:**319-324.
16. **Heil EL, Daniels LM, Long DM, Rodino KG, Weber DJ, Miller MB.** 2012. Impact of a rapid peptide nucleic acid fluorescence in situ hybridization assay on treatment of Candida infections. *Am J Health Syst Pharm* **69:**1910-1914.
17. **Herrera L, Culbreath K, Dehority W.** 2019. Impact of Matrix-Assisted Laser Desorption/Ionization Time-of-Flight for the Identification of Gram-Positive Bloodstream Pathogens without Antimicrobial Stewardship Intervention in Hospitalized Children. *J Pediatr Infect Dis* **14:**144-148.
18. **Holtzman C, Whitney D, Barlam T, Miller NS.** 2011. Assessment of impact of peptide nucleic acid fluorescence in situ hybridization for rapid identification of coagulase-negative staphylococci in the absence of antimicrobial stewardship intervention. *J Clin Microbiol* **49:**1581-1582.
19. **Koncelik DL, Hernandez J.** 2016. The Impact of Implementation of Rapid QuickFISH Testing for Detection of Coagulase-Negative Staphylococci at a Community-Based Hospital. *Am J Clin Pathol* **145:**69-74.
20. **Lockwood AM, Perez KK, Musick WL, Ikwuagwu JO, Attia E, Fasoranti OO, Cernoch PL, Olsen RJ, Musser JM.** 2016. Integrating Rapid Diagnostics and Antimicrobial Stewardship in Two Community Hospitals Improved Process Measures and Antibiotic Adjustment Time. *Infect Control Hosp Epidemiol* **37:**425-432.
21. **MacVane SH, Hurst JM, Boger MS, Gnann JW, Jr.** 2016. Impact of a rapid multiplex polymerase chain reaction blood culture identification technology on outcomes in patients with vancomycin-resistant Enterococcal bacteremia. *Infect Dis (Lond)* **48:**732-737.
22. **MacVane SH, Nolte FS.** 2016. Benefits of Adding a Rapid PCR-Based Blood Culture Identification Panel to an Established Antimicrobial Stewardship Program. *J Clin Microbiol* **54:**2455-2463.
23. **Magarifuchi H, Hamada Y, Oho M, Kusaba K, Urakami T, Aoki Y.** 2018. Clinical utility of direct application of matrix-assisted laser desorption ionization time-of-flight mass spectrometry and rapid disk diffusion test in presumptive antimicrobial therapy for bacteremia. *J Infect Chemother* **24:**881-886.
24. **Nagel JL, Huang AM, Kunapuli A, Gandhi TN, Washer LL, Lassiter J, Patel T, Newton DW.** 2014. Impact of antimicrobial stewardship intervention on coagulase-negative Staphylococcus blood cultures in conjunction with rapid diagnostic testing. *J Clin Microbiol* **52:**2849-2854.
25. **Neuner EA, Pallotta AM, Lam SW, Stowe D, Gordon SM, Procop GW, Richter SS.** 2016. Experience With Rapid Microarray-Based Diagnostic Technology and Antimicrobial Stewardship for Patients With Gram-Positive Bacteremia. *Infect Control Hosp Epidemiol* **37:**1361-1366.
26. **Nguyen DT, Yeh E, Perry S, Luo RF, Pinsky BA, Lee BP, Sisodiya D, Baron EJ, Banaei N.** 2010. Real-time PCR testing for mecA reduces vancomycin usage and length of hospitalization for patients infected with methicillin-sensitive staphylococci. *J Clin Microbiol* **48:**785-790.
27. **Nicolsen NC, LeCroy N, Alby K, Martin KE, Laux J, Lin FC, Daniels L, Weber DJ, Miller MB.** 2013. Clinical outcomes with rapid detection of methicillin-resistant and methicillin-susceptible Staphylococcus aureus isolates from routine blood cultures. *J Clin Microbiol* **51:**4126-4129.
28. **Niwa T, Yonetamari J, Hayama N, Fujibayashi A, Ito-Takeichi S, Suzuki K, Ohta H, Niwa A, Tsuchiya M, Yamamoto M, Hatakeyama D, Hayashi H, Obara M, Sugiyama T, Baba H, Suzuki A, Murakami N.** 2019. Clinical impact of matrix-assisted laser desorption ionization-time of flight mass spectrometry combined with antimicrobial stewardship interventions in patients with bloodstream infections in a Japanese tertiary hospital. *Int J Clin Pract* **73:**e13332.
29. **Pardo J, Klinker KP, Borgert SJ, Butler BM, Giglio PG, Rand KH.** 2016. Clinical and economic impact of antimicrobial stewardship interventions with the FilmArray blood culture identification panel. *Diagn Microbiol Infect Dis* **84:**159-164.
30. **Perez KK, Olsen RJ, Musick WL, Cernoch PL, Davis JR, Land GA, Peterson LE, Musser JM.** 2013. Integrating rapid pathogen identification and antimicrobial stewardship significantly decreases hospital costs. *Arch Pathol Lab Med* **137:**1247-1254.
31. **Romero-Gomez MP, Cendejas-Bueno E, Garcia Rodriguez J, Mingorance J.** 2017. Impact of rapid diagnosis of Staphylococcus aureus bacteremia from positive blood cultures on patient management. *Eur J Clin Microbiol Infect Dis* **36:**2469-2473.
32. **Roshdy DG, Tran A, LeCroy N, Zeng D, Ou FS, Daniels LM, Weber DJ, Alby K, Miller MB.** 2015. Impact of a rapid microarray-based assay for identification of positive blood cultures for treatment optimization for patients with streptococcal and enterococcal bacteremia. *J Clin Microbiol* **53:**1411-1414.
33. **Seo SK, Gedrimaite Z, Paskovaty A, Seier K, Morjaria S, Cohen N, Riedel E, Tang YW, Babady NE.** 2018. Impact of QuickFISH in addition to antimicrobial stewardship on vancomycin use and resource utilization in cancer patients with coagulase-negative staphylococcal blood cultures. *Clin Microbiol Infect* **24:**1339 e1337-1339 e1312.
34. **Sothoron C, Ferreira J, Guzman N, Aldridge P, McCarter YS, Jankowski CA.** 2015. A Stewardship Approach To Optimize Antimicrobial Therapy through Use of a Rapid Microarray Assay on Blood Cultures Positive for Gram-Negative Bacteria. *J Clin Microbiol* **53:**3627-3629.
35. **Suzuki H, Hitomi S, Yaguchi Y, Tamai K, Ueda A, Kamata K, Tokuda Y, Koganemaru H, Kurihara Y, Ishikawa H, Yanagisawa H, Yanagihara K.** 2015. Prospective intervention study with a microarray-based, multiplexed, automated molecular diagnosis instrument (Verigene system) for the rapid diagnosis of bloodstream infections, and its impact on the clinical outcomes. *J Infect Chemother* **21:**849-856.
36. **Tseng AS, Kasule SN, Rice F, Mi L, Chan L, Seville MT, Grys TE.** 2018. Is It Actionable? An Evaluation of the Rapid PCR-Based Blood Culture Identification Panel on the Management of Gram-Positive and Gram-Negative Blood Stream Infections. *Open Forum Infect Dis* **5:**ofy308.
37. **Turner RB, Lalikian K, Fry M, Schwartz J, Chan D, Won R.** 2017. Impact of rapid identification of Staphylococcus aureus bloodstream infection without antimicrobial stewardship intervention on antibiotic optimization and clinical outcomes. *Diagn Microbiol Infect Dis* **89:**125-130.
38. **Vlek AL, Bonten MJ, Boel CH.** 2012. Direct matrix-assisted laser desorption ionization time-of-flight mass spectrometry improves appropriateness of antibiotic treatment of bacteremia. *PLoS One* **7:**e32589.
39. **Walker T, Dumadag S, Lee CJ, Lee SH, Bender JM, Cupo Abbott J, She RC.** 2016. Clinical Impact of Laboratory Implementation of Verigene BC-GN Microarray-Based Assay for Detection of Gram-Negative Bacteria in Positive Blood Cultures. *J Clin Microbiol* **54:**1789-1796.

**Fig. S3**

1. **Forrest GN, Mehta S, Weekes E, Lincalis DP, Johnson JK, Venezia RA.** 2006. Impact of rapid *in situ* hybridization testing on coagulase-negative staphylococci positive blood cultures. *J Antimicrob Chemother* **58:**154-158.

**Fig. S4**

1. **Banerjee R, Teng CB, Cunningham SA, Ihde SM, Steckelberg JM, Moriarty JP, Shah ND, Mandrekar JN, Patel R.** 2015. Randomized Trial of Rapid Multiplex Polymerase Chain Reaction-Based Blood Culture Identification and Susceptibility Testing. *Clin Infect Dis* **61:**1071-1080.
2. **Beuving J, Wolffs PF, Hansen WL, Stobberingh EE, Bruggeman CA, Kessels A, Verbon A.** 2015. Impact of same-day antibiotic susceptibility testing on time to appropriate antibiotic treatment of patients with bacteraemia: a randomised controlled trial. *Eur J Clin Microbiol Infect Dis* **34:**831-838.
3. **Bhat BV, Prasad P, Ravi Kumar VB, Harish BN, Krishnakumari K, Rekha A, Manjunath G, Adhisivam B, Shruthi B.** 2016. Syndrome Evaluation System (SES) *versus* Blood Culture (BACTEC) in the Diagnosis and Management of Neonatal Sepsis - A Randomized Controlled Trial. *Indian J Pediatr* **83:**370-379.
4. **Bunsow E, Vecchio MG, Sanchez C, Munoz P, Burillo A, Bouza E.** 2015. Improved Sepsis Alert With a Telephone Call From the Clinical Microbiology Laboratory: A Clinical Trial. *Medicine (Baltimore)* **94:**e1454.
5. **Cattoir V, Merabet L, Djibo N, Rioux C, Legrand P, Girou E, Lesprit P.** 2011. Clinical impact of a real-time PCR assay for rapid identification of Staphylococcus aureus and determination of methicillin resistance from positive blood cultures. *Clin Microbiol Infect* **17:**425-431.
6. **Cosgrove SE, Li DX, Tamma PD, Avdic E, Hadhazy E, Wakefield T, Gherna M, Carroll KC.** 2016. Use of PNA FISH for blood cultures growing Gram-positive cocci in chains without a concomitant antibiotic stewardship intervention does not improve time to appropriate antibiotic therapy. *Diagn Microbiol Infect Dis* **86:**86-92.
7. **Idelevich EA, Silling G, Niederbracht Y, Penner H, Sauerland MC, Tafelski S, Nachtigall I, Berdel WE, Peters G, Becker K, Molecular Diagnostics of Sepsis Study G.** 2015. Impact of multiplex PCR on antimicrobial treatment in febrile neutropenia: a randomized controlled study. *Med Microbiol Immunol* **204:**585-592.
8. **Neuberger A, Oren I, Sprecher H.** 2008. Clinical impact of a PCR assay for rapid identification of Klebsiella pneumoniae in blood cultures. *J Clin Microbiol* **46:**377-379.
9. **Rodrigues C, Siciliano RF, Filho HC, Charbel CE, de Carvalho Sarahyba da Silva L, Baiardo Redaelli M, de Paula Rosa Passetti AP, Franco MRG, Rossi F, Zeigler R, De Backer D, Franco RA, de Almeida JP, Rizk SI, Fukushima JT, Landoni G, Uip DE, Hajjar LA, Strabelli TMV.** 2019. The effect of a rapid molecular blood test on the use of antibiotics for nosocomial sepsis: a randomized clinical trial. *J Intensive Care* **7:**37.
10. **Tafelski S, Nachtigall I, Adam T, Bereswill S, Faust J, Tamarkin A, Trefzer T, Deja M, Idelevich EA, Wernecke KD, Becker K, Spies C, Molecular Diagnostics of Sepsis Study G.** 2015. Randomized controlled clinical trial evaluating multiplex polymerase chain reaction for pathogen identification and therapy adaptation in critical care patients with pulmonary or abdominal sepsis. *J Int Med Res* **43:**364-377.
