# Supplementary material for: The American Society for Microbiology’s evidence-based laboratory medicine practice guidelines for the diagnosis of bloodstream infections using rapid tests: a systematic review and meta-analysis
Source: Clin Microbiol Rev. 2025 Jun 16;38(3):e00137-24. doi: 10.1128/cmr.00137-24 (PMC12424361; doi:10.1128/cmr.00137-24)
Supplement: Text S1 — Executive summary and recommendations. [file cmr.00137-24-s0010.docx]

BOX 1. Executive summary and recommendations

Identifying pathogens and corresponding antimicrobial susceptibility results optimizes care for patients with bloodstream infections (BSIs). Several tests, including nucleic acids amplification tests (NAAT), hybridization tests, and matrix-assisted laser desorption ionization time-of-flight mass spectrometry, rapidly identify common bacteria and yeast directly from a positive blood culture. Some molecular methods include genetic markers associated with antibiotic resistance. Because strategies used for rapid diagnostic tests (RDTs) vary by laboratory, the American Society for Microbiology assembled an expert panel to review the existing literature describing the effectiveness of RDTs in decreasing the time-to-targeted therapy for hospitalized patients with BSIs (1). The review was published in 2016, but no recommendations were made due to insufficient evidence. With the increased availability and broader use of rapid diagnostics, a second expert panel was convened to assess the evidence that RDTs improve outcomes in adult and pediatric patients hospitalized with BSIs, provided that active communication occurs. Because of differences in global healthcare infrastructures, the published literature varies in the way active communication is described. Some examples include phone calls or texts to a provider or active communication (voice or digital) to pharmacists or microbiologists associated with antimicrobial stewardship programs (2) (https://www.cdc.gov/antibiotic-use/hcp/core-elements/hospital.html), diagnostic stewardship programs (3), or other active methods determined in collaboration with healthcare providers and stakeholders. Based on the results of a scoping review (4), the panel evaluated the effectiveness of rapid diagnostics and developed recommendations to improve three clinical outcomes: time-to-targeted therapy, length of hospital stay, and mortality.

SUMMARY OF RECOMMENDATIONS

*Recommendations for the use of rapid diagnostic tests to decrease time-to-targeted therapy (TTT)*

**Recommendation 1:** To decrease **TTT** in patients with a positive blood culture, the panel **recommends** that clinical laboratories implement rapid diagnostic tests *(Evidence quality: Moderate; Recommendation strength: Strong)* **in combination with** a comprehensive plan to actively communicate the actionable test result(s) *(Evidence quality: Strong; Recommendation strength: Strong)*.

**Remarks:**

- Most studies evaluating the impact of diagnostic tests on clinical outcomes use a “Before/After” design that generally falls lower on the strength of the evidence scale. However, the panel considered several elements, including the number of studies and the certainty of the evidence when making the recommendation.
- The panel does NOT recommend using rapid diagnostics tests alone, without active communication, to improve TTT.
- The data to assess the impact of rapid diagnostic tests in separate patient populations (e.g., immunocompromised vs immunocompetent hosts) was limited.
- There was insufficient evidence to assess the impact of different rapid diagnostic test methods (e.g., NAAT vs MALDI-TOF MS).

*Recommendations for the use of rapid diagnostics to decrease length of stay (LOS)*

**Recommendation 2:** To decrease the **hospital LOS** in hospitalized patients with a positive blood culture, the panel **recommends** that clinical laboratories implement rapid diagnostic tests *(Evidence quality: low; Recommendation strength: Strong)* **in combination with** a comprehensive plan to actively communicate the actionable test result (s) *(Evidence quality: Moderate; Recommendation strength: Strong)*.

**Remarks:**

- Most studies evaluating the impact of diagnostic tests on clinical outcomes use Before/After designs that generally fall lower on the strength of the evidence scale. However, the panel considered several elements, including the number of studies and the certainty of the evidence when making the recommendation.
- The panel considered that the assessment of LOS varied in different hospitals, whose discharge processes vary and impact LOS regardless of the laboratory’s use of rapid testing. Given the trend towards lower LOS (impacted by discharge protocols), the panel decided to weigh the data more heavily and make a strong recommendation.
- The panel does NOT recommend using rapid diagnostics tests alone, without active communications, to decrease hospital LOS. There was insufficient evidence to assess the impact of different rapid diagnostic test methods (e.g., NAAT vs MALDI-TOF MS).

**Recommendation 3:** To decrease the **LOS in an Intensive Care Unit (ICU)** for patients with a positive blood culture, the panel **suggests** that clinical laboratories implement rapid diagnostic tests *(Evidence quality: Moderate; Recommendation strength: Strong)* **with OR without** the healthcare organization implementing a comprehensive plan to actively communicate the actionable test result(s) *(Evidence quality: Moderate; Recommendation strength: Strong)*.

**Remarks:**

- Most studies evaluating the impact of diagnostic tests on clinical outcomes use a Before/After design that generally falls lower on the strength of the evidence scale. However, the panel considered several elements, including the number of studies and the certainty of the evidence when making the recommendation.
- The ICU LOS was lower using rapid diagnostics with or without active communication, but the overall number of studies was smaller than that of all hospital LOS studies. The panel could not recommend guidance for or against using rapid diagnostics to decrease ICU LOS but suggests rapid diagnostics as best practice in combination with active communication. There was insufficient evidence to assess the impact of different rapid diagnostic test methods (e.g., NAAT vs MALDI-TOF MS).

**Recommendation 4:** To decrease the **infection-related LOS** for patients with a positive blood culture, the panel **recommends** that clinical laboratories implement rapid diagnostic tests *(Evidence quality: Moderate; Recommendation strength: Strong)* **in combination with** a comprehensive plan to actively communicate the actionable test result(s) *(Evidence quality: Moderate; Recommendation strength: Strong)*.

**Remarks:**

- Most studies evaluating the impact of diagnostic tests on clinical outcomes use Before/After designs that generally fall lower on the strength of the evidence scale. However, the panel considered several elements, including the number of studies and the certainty of the evidence when making the recommendation.
- The panel recommends against using a “test only” approach without concomitant enhancements to actively communicate actionable test results. Given that only one study examined the effect of a “test only” intervention, our inference here is less certain than with hospital or ICU LOS. However, drawing on the Bradford-Hill criteria for causal inference, the principle of “analogy” suggests that, given additional data, we should expect the same causal relations to exist for infection-related LOS as with hospital or ICU LOS **(5)**.

*Recommendations for the use of rapid diagnostics to decrease mortality*

**Recommendation 5:** To decrease the **30-day mortality** in patients with a positive blood culture, the panel **recommends** that clinical laboratories implement rapid diagnostic tests *(Evidence quality: Low; Recommendation strength: Moderate)* **in combination with a** comprehensive plan to actively communicate the actionable test result(s) *(Evidence quality: Low; Recommendation strength: Moderate)*.

**Remarks:**

- Most studies evaluating the impact of diagnostic tests on clinical outcomes use a Before/After design that generally falls lower on the strength of the evidence scale. However, the panel considered several elements, including the number of studies and the certainty of the evidence when making the recommendation.
- The overall impact of rapid diagnostics tests on 30-day mortality varied by study type, with a more significant decrease reported in observational studies.
- The panel does NOT recommend using rapid diagnostics tests alone to decrease 30-day mortality.

**Recommendation 6:** To decrease **non-specific mortality** of patients with a positive blood culture, the panel **suggests** that clinical laboratories implement rapid diagnostic tests *(Evidence quality: Low; Recommendation strength: Weak)* **in combination with** a comprehensive plan to actively communicate the actionable test result(s) *(Evidence quality: Low; Recommendation strength: Weak)*.

**Remarks:**

- Most studies evaluating the impact of diagnostic tests on clinical outcomes use a Before/After design that generally falls lower on the strength of the evidence scale. However, the panel considered several elements, including the number of studies and the certainty of the evidence when making the recommendation.
- The overall impact of rapid diagnostics tests on non-specific mortality varied by study type (i.e., observational vs. controlled trials). While the results from the non-RCT studies (very low confidence level) were equivocal, the results from the two controlled trials provided high confidence that implementation of a rapid test combined with active communication could result in as many as 134 fewer deaths to as few as 7 fewer deaths per 1000 patients.
- The panel could not make recommendations for or against using rapid diagnostics with or without active communication to decrease non-specific mortality but suggested using rapid diagnostics in combination with active communication as a best practice recommendation.

**Recommendation 7:** To decrease the **in-hospital mortality** in patients with a positive blood culture, the panel **suggests** that clinical laboratories implement rapid diagnostics *(Evidence quality: Low; Recommendation strength: Low)* **in combination with** a comprehensive plan to actively communicate the actionable test result(s) *(Evidence quality: Low; Recommendation strength: Low)*.

**Remarks:**

- Most studies evaluated the impact of diagnostic tests on clinical outcomes using Before/After designs that generally fell lower on the strength of the evidence rank. However, the panel considered several elements, including the number of studies and the certainty of the evidence when making the recommendation.
- There was an overall trend towards lower in-hospital mortality, but the difference was not statistically significant.
- The panel could not make recommendations for or against using rapid diagnostics with or without active communication to decrease hospital mortality, but suggested using rapid diagnostics in combination with active communication as a best practice recommendation. Hospital mortality is likely to be a multifactorial variable, and many of the studies were not sufficiently statistically powered to assess the impact of rapid diagnostics on the many individual factors that impact mortality.

**Recommendation 8:** To decrease **infection-related mortality** in patients with a positive blood culture, the panel **suggests** that clinical laboratories implement rapid diagnostic tests *(Evidence quality: Low; Recommendation strength: Very low)* **in combination** with a comprehensive plan to actively communicate the actionable test result(s) *(Evidence quality: Low; Recommendation strength: Very low)*.

**Remarks:**

- Only one low-risk Before/After study was identified; it utilized a comprehensive intervention (rapid test + active communication), which was available for the analysis. However, the panel considered similar patterns found in other mortality outcome analyses. While this evidence is indirect, it bolsters the inference that using a rapid test with an effective communication protocol may have similar beneficial results.

REFERENCES

1. **Buehler SS, Madison B, Snyder SR, Derzon JH, Cornish NE, Saubolle MA, Weissfeld AS, Weinstein MP, Liebow EB, Wolk DM.** 2016. Effectiveness of Practices To Increase Timeliness of Providing Targeted Therapy for Inpatients with Bloodstream Infections: a Laboratory Medicine Best Practices Systematic Review and Meta-analysis. *Clin Microbiol Rev* **29:**59-103.

2. **Dellit TH, Owens RC, McGowan JE, Gerding DN, Weinstein RA, Burke JP, Huskins WC, Paterson DL, Fishman NO, Carpenter CF, Brennan PJ, Billeter M, Hooton TM.** 2007. Infectious Diseases Society of America and the Society for Healthcare Epidemiology of America Guidelines for Developing an Institutional Program to Enhance Antimicrobial Stewardship. *Clinical Infectious Diseases* **44:**159-177.

3. **Patel R, Fang FC.** 2018. Diagnostic Stewardship: Opportunity for a Laboratory-Infectious Diseases Partnership. *Clin Infect Dis* **67:**799-801.

4. **Rubinstein ML, Wolk DM, Babady NE, Johnson JK, Atkinson B, Makim R, Parrott JS.** 2021. Mapping the Evidence on Rapid Diagnosis of Bloodstream Infections: A Scoping Review. *J Appl Lab Med* **6:**1012-1024.

5. **Shimonovich M, Pearce A, Thomson H, Keyes K, Katikireddi SV.** 2021. Assessing causality in epidemiology: revisiting Bradford Hill to incorporate developments in causal thinking. *Eur J Epidemiol* **36:**873-887.
